# Supplementary material for: Smart-watch-programmed green-light-operated percutaneous control of therapeutic transgenes
Source: Nat Commun. 2021 Jun 7;12:3388. doi: 10.1038/s41467-021-23572-4 (PMC8184832; doi:10.1038/s41467-021-23572-4)
Supplement: Supplementary file 1 — Supplementary Information [file 41467_2021_23572_MOESM1_ESM.pdf]

Supplementary Information

Smart-Watch-Programmed Green-Light-Operated Percutaneous Control of Therapeutic Transgenes

Maysam Mansouri[1], Marie-Didiée Hussherr[1], Tobias Strittmatter[1], Peter Buchmann[1], Shuai Xue[1], Gieri Camenisch[1], and Martin Fussenegger[1,2*]

[1] Department of Biosystems Science and Engineering, ETH Zurich, Basel, Switzerland.
[2] Faculty of Science, University of Basel, Mattenstrasse 26, CH-4058, Basel, Switzerland.

*Corresponding author. E-mail: fussenegger@bsse.ethz.ch

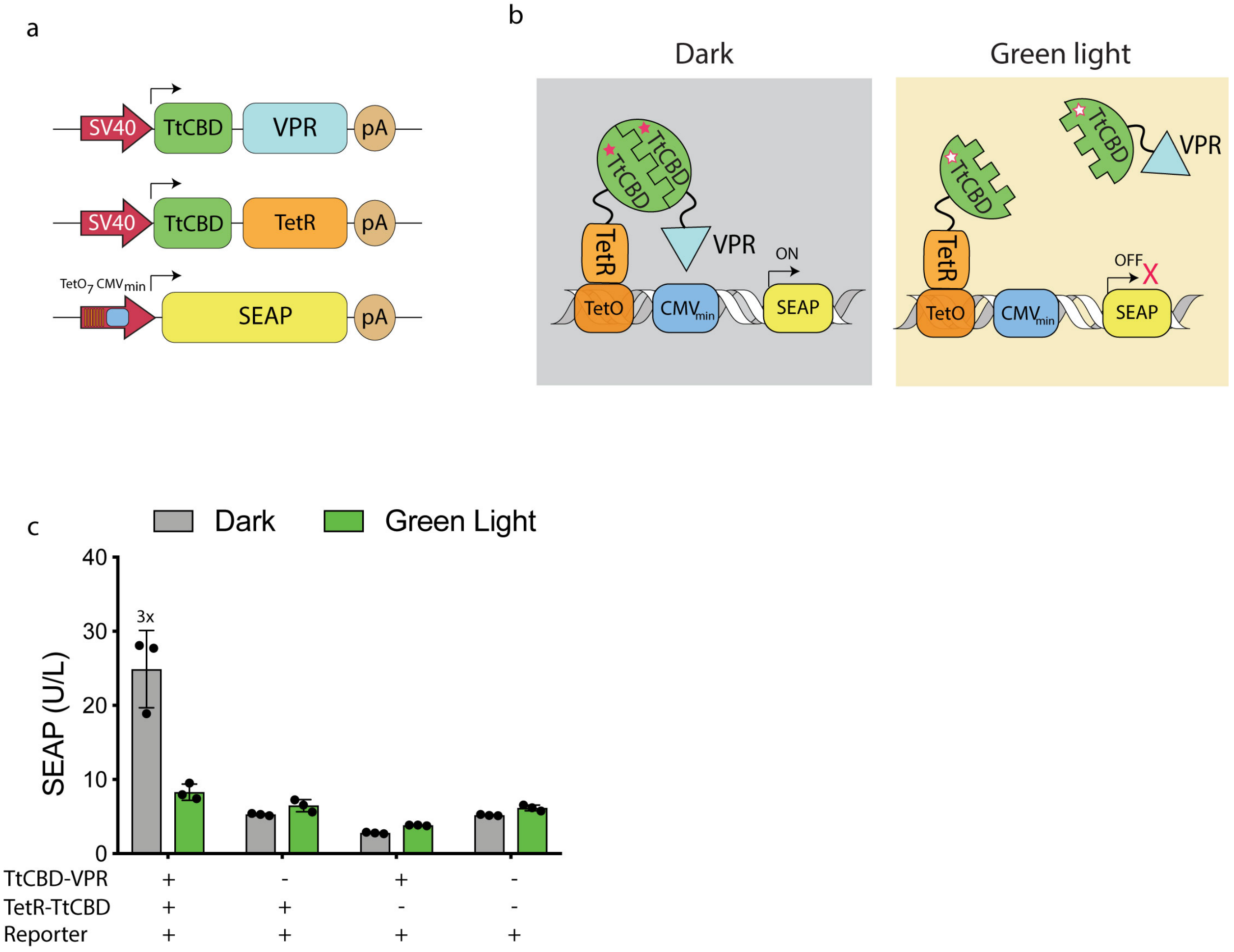

Supplementary Figure 1 | Design of green-light-mediated transgene suppression (Green OFF) system in mammalian cells. a, The DNA constructs used for the Green OFF system. The Green OFF circuit is based on interactions of a TtCBD-VPR (pMMZ295; P_{SV40}-TtCBD-VPR-pA) and TtCBD-TetR (pMMZ271; P_{SV40}-TtCBD-TetR-pA), leading to expression of the SEAP reporter gene from the synthetic promoter P_{TET} (pTS1017; $P_{TetO7\text{-}hCMVmin}$-SEAP-pA). **b**, Schematic representation of the Green OFF system in the dark and upon illumination with green light. In the presence of $AdoB_{12}$ (filled asterisk) TtCBD-VPR interacts with TtCBD-TetR in the dark. This complex binds to P_{TET} on a synthetic promoter to initiate expression of the reporter gene SEAP. Dissociation of the complex upon green light illumination reverts the promoter to an uninduced state. **c**, Dependency of the Green OFF system on the presence (+) or absence (-) of each component was studied by quantification of the reporter SEAP in the supernatant of HEK293T cells transfected with the indicated plasmids. Cells were induced with pulsed green light (545 nm; 15 sec ON/45 sec OFF and 88 $\mu W/cm^2$) for 48 h. Bars represent the mean ± s.d. (n = 3), and 3x above the left bar indicate a 3-fold difference of reporter (SEAP) expression versus the corresponding green-light-on experiment. Source data are provided as a Source Data file.

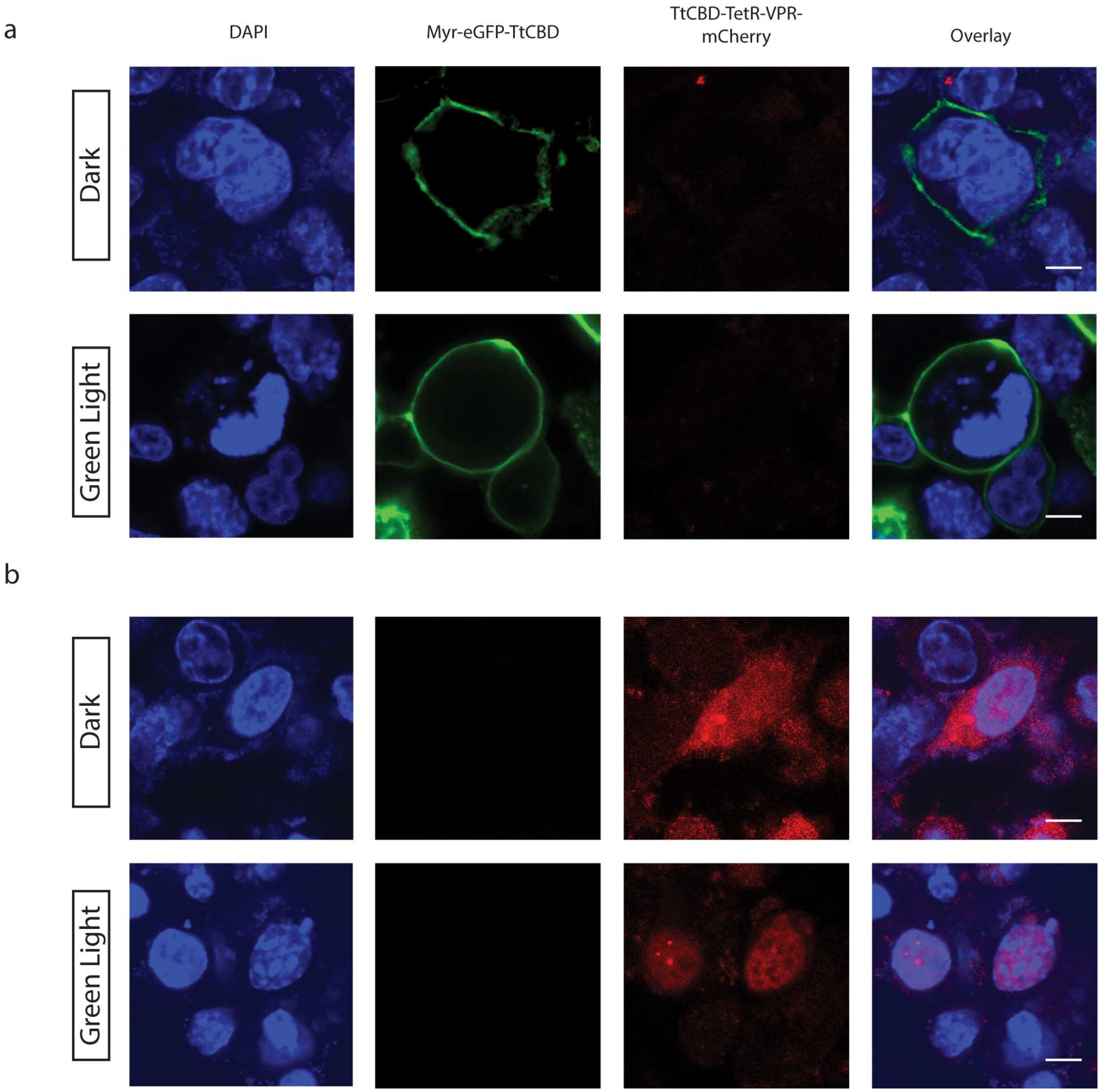

Supplementary Figure 2 | Microscopy analysis of the Glow Control components. HEK293T cells were transfected with either (**a**) pMMZ351 (P_{SV40}-Myr-TtCBD-GFP-pA) or (**b**) pMMZ429 (P_{SV40}-TtCBD-TetR-VPR-mCherry-pA) and induced with green light (545 nm; 12 h/day, 15 sec ON and 45 sec OFF and 88 μW/cm^2) for 48 h. Control groups were kept in dark. Cells were fixed with 4% PFA and stained with DAPI. Scale bars, 10 μm. A representative image of three replicates from each group is shown.

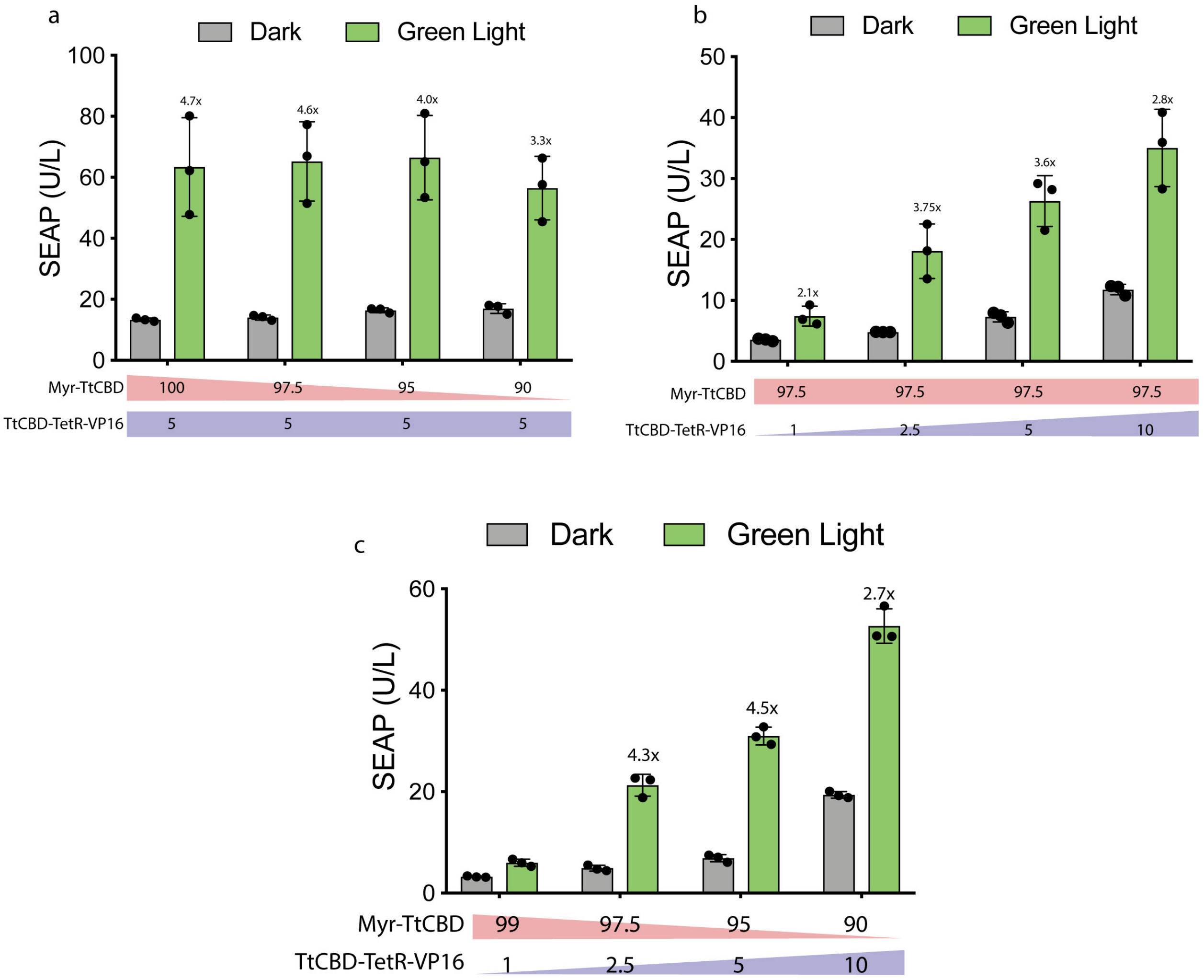

Supplementary Figure 3 | Optimization of plasmid ratio in Glow Control system. The Glow Control system plasmids (pMMZ269; P_{SV40}-Myr-TtCBD-pA, and transactivator pMMZ272; P_{SV40}-TtCBD-TetR-VP16-pA) were co-transfected with a constant amount of the P_{TET} reporter (pTS1017; $P_{TetO7\text{-}hCMVmin}$-SEAP-pA). Different ratios of Myr-TtCBD (constant amount in **b** and reducing in **a** and **c**) to transactivator (constant amount in **a** and increasing in **b** and **c**) were applied to optimize SEAP expression in the Glow Control system. Numbers below are amounts of DNA (ng/well) used for co-transfection in HEK293T cells seeded in 96-well plates. Transfected cells were illuminated with green light (545 nm; 15 sec ON/45 sec OFF and 88 μW/cm^2 for 12 h/day) and SEAP expression was measured 48 h after first illumination. Control cells were kept in the dark. Bars represent the mean ± s.d. (n = 3), and numbers above the bars indicate fold changes of reporter (SEAP) expression compared to the corresponding dark control. Source data are provided as a Source Data file.

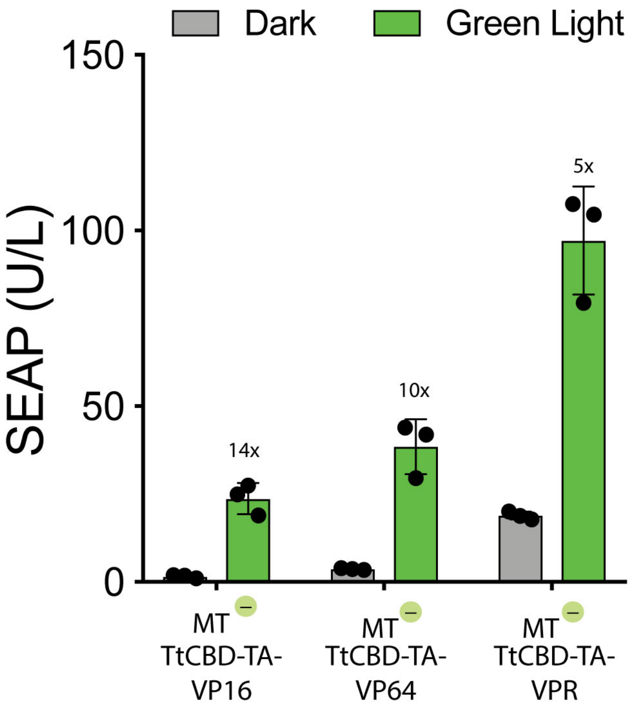

Supplementary Figure 4 | Comparison between different TtCBD-transactivator variants in improved version of Glow Control system. HEK293T cells were transfected with negatively charged Myr-TtCBD (pMMZ284; P_{SV40}-Myr-TtCBD-nGFP-pA), P_{TET} reporter and either of different TtCBD-transactivator (TtCBD-TA) variants (pMMZ272; P_{SV40}-TtCBD-TetR-VP16-pA, pMMZ304; P_{SV40}-TtCBD-TetR-VP64-pA, and pMMZ273; P_{SV40}-TtCBD-TetR-VPR-pA). Cells were stimulated with pulsed green light (545 nm; 15 sec ON/45 sec OFF and 88 μW/cm^2) for 12 h/day and SEAP expression was measured at 48 h after the start of illumination. Control cells were incubated in the dark. Bars represent mean ± s.d. (n = 3), and numbers above the bars indicate fold changes of reporter (SEAP) expression compared to the corresponding dark control. Source data are provided as a Source Data file.

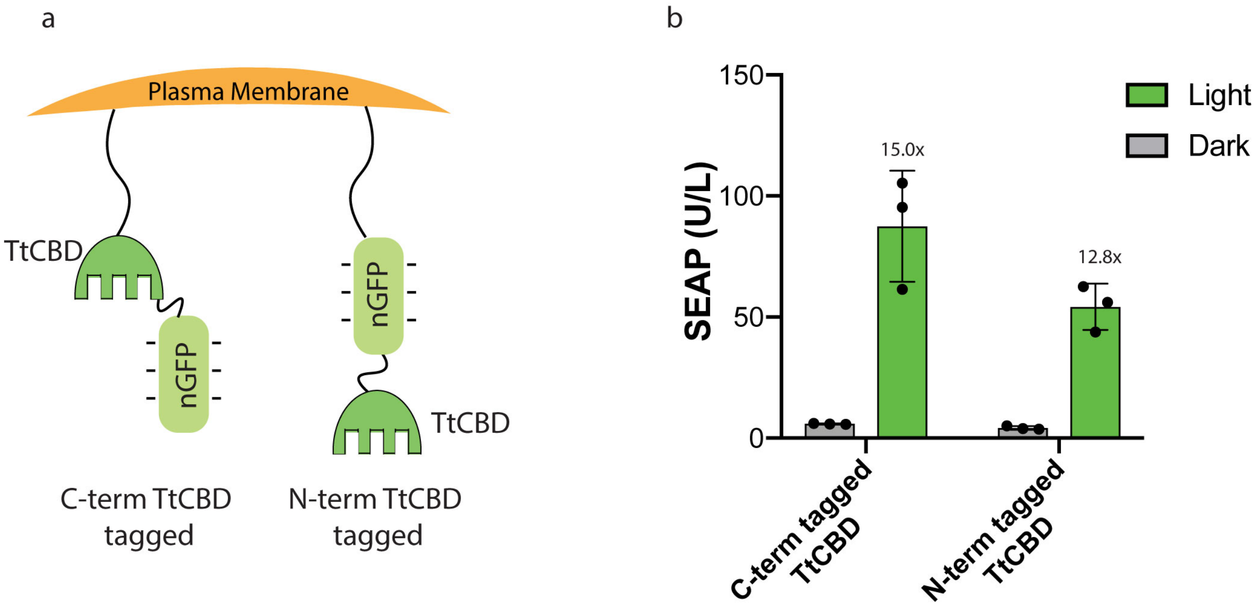

Supplementary Figure 5 | Comparison of N-terminal or C-terminal tagging of TtCBD in improved version of Glow Control. a, Schematic representation of plasma membrane-anchored TtCBD tagged C-terminally (C-term) or N-terminally (N-term) with negatively supercharged GFP. **b**, HEK293T cells were co-transfected with pMMZ272 (P_{SV40}-TtCBD-TetR-VP16-pA), pTS1017 (TetO7-$P_{hCMVmin}$-SEAP-pA) and either pMMZ284 (P_{SV40}-Myr-TtCBD-nGFP-pA) or pMMZ410 (P_{SV40}-Myr-nGFP-TtCBD-pA). Cells were illuminated with green light (545 nm; 15 sec ON/45 sec OFF and 88 μW/cm^2 for 12 h/day) and SEAP expression was measured 48h after first illumination. Control cells kept in dark. Bars represent the mean ± s.d. (n = 3), and numbers above the bars indicate fold changes of reporter (SEAP) expression compared to the corresponding dark control. Source data are provided as a Source Data file.

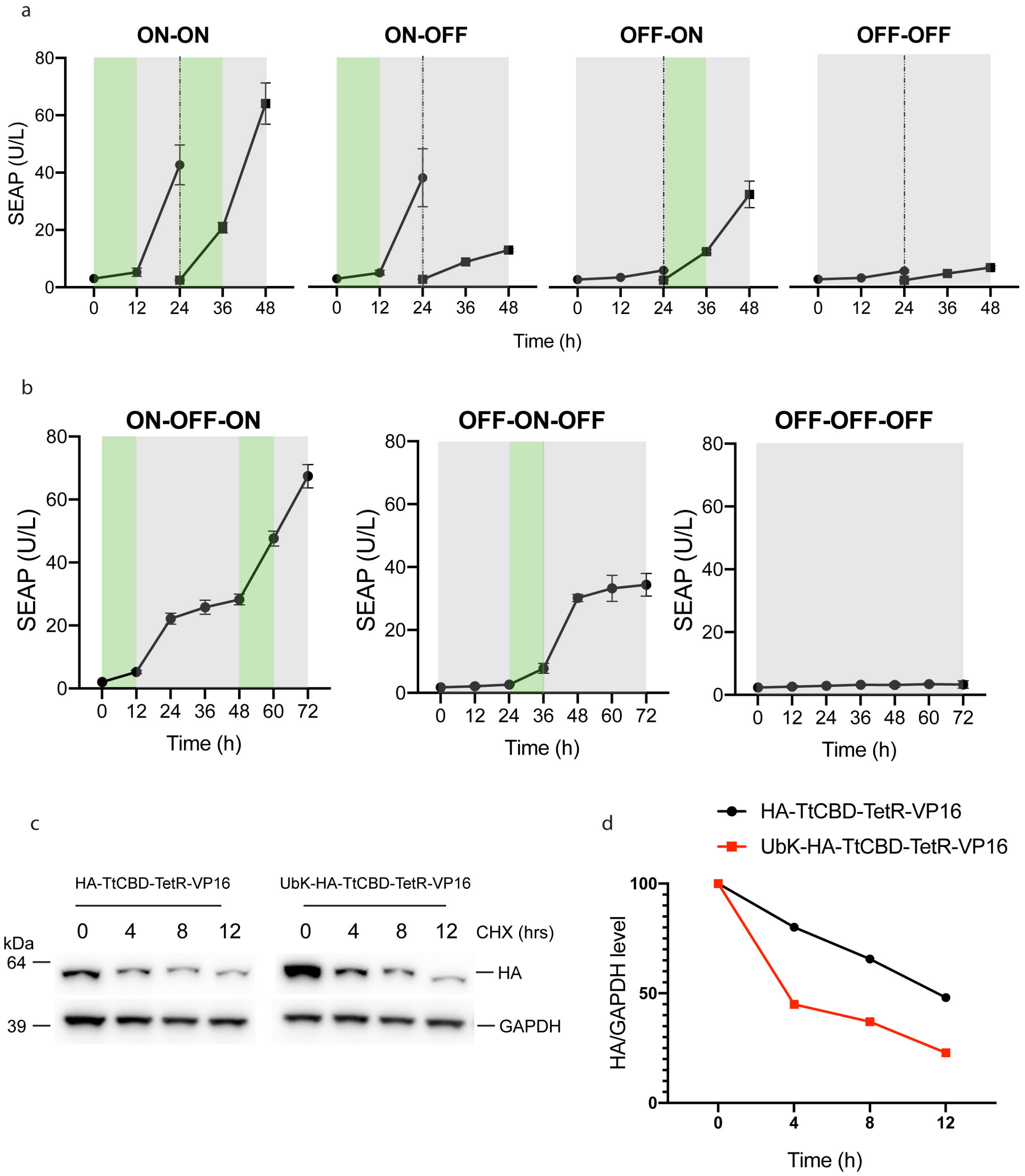

Supplementary Figure 6 | In vitro reversibility of the Glow Control system and half-life of TtCBD protein. a, Extended figures related to Figure 3c. HEK293T cells were illuminated with pulsed green light (ON, indicated by green box) or kept in the dark (OFF, indicated by grey box) and SEAP levels were profiled every 12 h for 48 h. After 24 h, the culture medium was changed and the cell density was adjusted to 5 x10^4 cells/well of a 96-well plate. The pattern of light/dark cycles is indicated above each plot. The values are mean ± s.d., n = 3 biological replicates. **b**, In

vitro reversibility of Glow Control in a continuous culture. Transfected HEK293T cells were illuminated with pulsed green light (ON; green box) or were kept in the dark (OFF; dark box) and 10 μl of supernatant (replaced by the same volume of fresh complete media) was taken at each indicated time point for SEAP measurement. The pattern of light/dark cycles is indicated above each plot. The values are mean ± s.d., n = 6 biological replicates. **c**, TtCBD-TA half-life measurement. HEK293T cells were transfected with TtCBD-TA (pMMZ516; P_{CMV}-HA-TtCBD-TetR-VP16-pA) or a degron-tagged version of TtCBD-TA (MMZ518; P_{CMV}-Ubk-HA-TtCBD-TetR-VP16-pA) and were cultured in presence of the protein synthesis inhibitor cycloheximide (CHX, 25 μg/mL). Protein half-life was measured by western blotting after the indicated times of treatment. A representative image of three replicates is shown. **d**, Western blot densitometric quantification of TtCBD-TA (black) and degron-tagged TtCBD-TA (red). Plotted TtCBD-TA and Ubk-TtCBD-TA values are normalized against the housekeeping gene GAPDH. Source data are provided as a Source Data file.

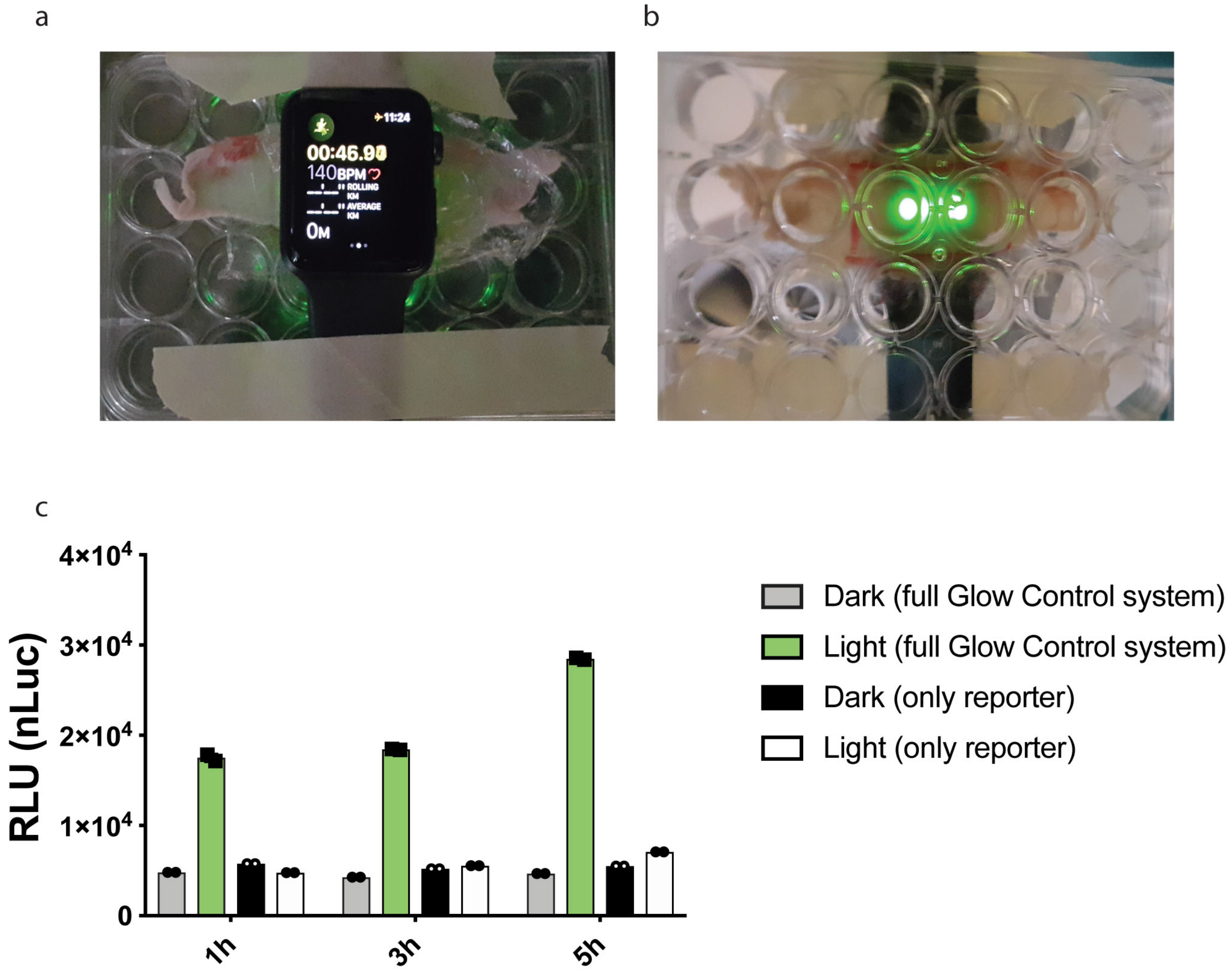

Supplementary Figure 7 | In vitro assessment of tissue penetration of green light emitted from an Apple Watch. a, View from above: HEK293T cells were co-transfected with pMMZ284/pMMZ272 and pLeo1403 ($P_{TetO7\text{-}CMVmin}$-nLuc-Fc-pA) or only pLeo1403 and illuminated with green light emitted from an Apple Watch for 1 h, 3 h and 5 h. A patch of mouse skin was placed on top of the cell culture plate between the watch and the cell culture plate. Control cells were kept in the dark. **b**, View from below. **c**, Quantification of produced SEAP in supernatant of HEK293T cells. Bars represent the mean ± s.d. (n = 3). Source data are provided as a Source Data file.

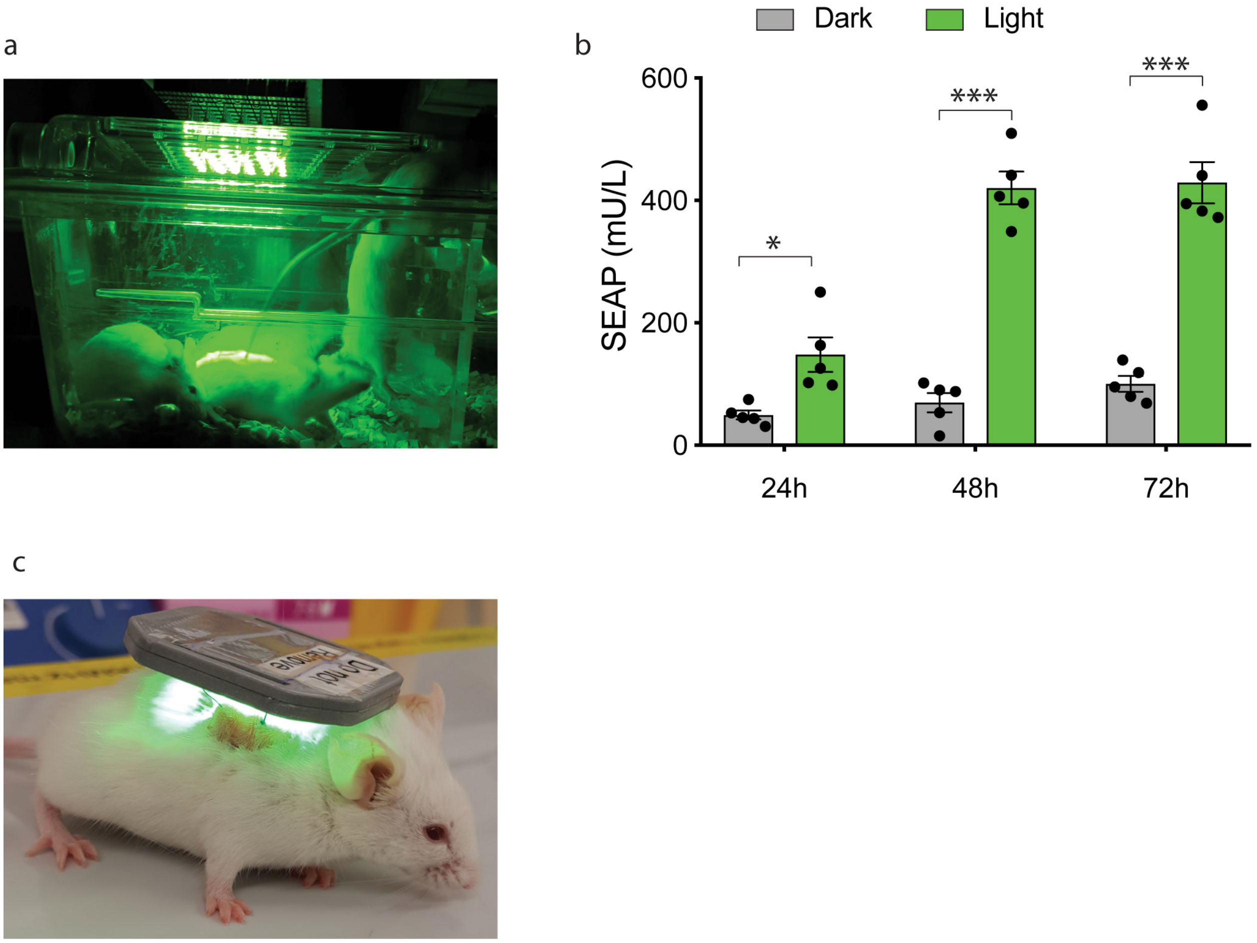

Supplementary Figure 8 | In vivo experimental set up. a, Light-shower-regulated SEAP expression in mice. 1x10^7 HEK293T cells transiently co-transfected with pMMZ284, pMMZ272 and pTS1017 were microencapsulated and subcutaneously implanted in RjOrl:SWISS (CD-1) mice. The mice were illuminated with green light (545 nm, 12 h/day, 15 sec ON/45 sec OFF, 300 μW/cm^2) from LEDs mounted on the ceiling of the cage. **b**, Quantification of SEAP levels in blood stream of light-shower-treated mice for three days. Values are mean ± SEM (n = 5), and statistical significance between the Light and Dark groups at the indicated time points were calculated using a two-tailed, paired Student's t-test. *, $p<0.05$; ***, $p<0.001$. **c**, Mice were illuminated with a programmable, smart-watch-mimicking LED patch (see Supplementary Figure 9) (545 nm, e.g., 3 h, constant or pulsed for 12 h/day, 15 sec ON/45 sec OFF, 150 μW/cm^2), which was fixed with sutures to the shaved back of the mice. Source data are provided as a Source Data file.

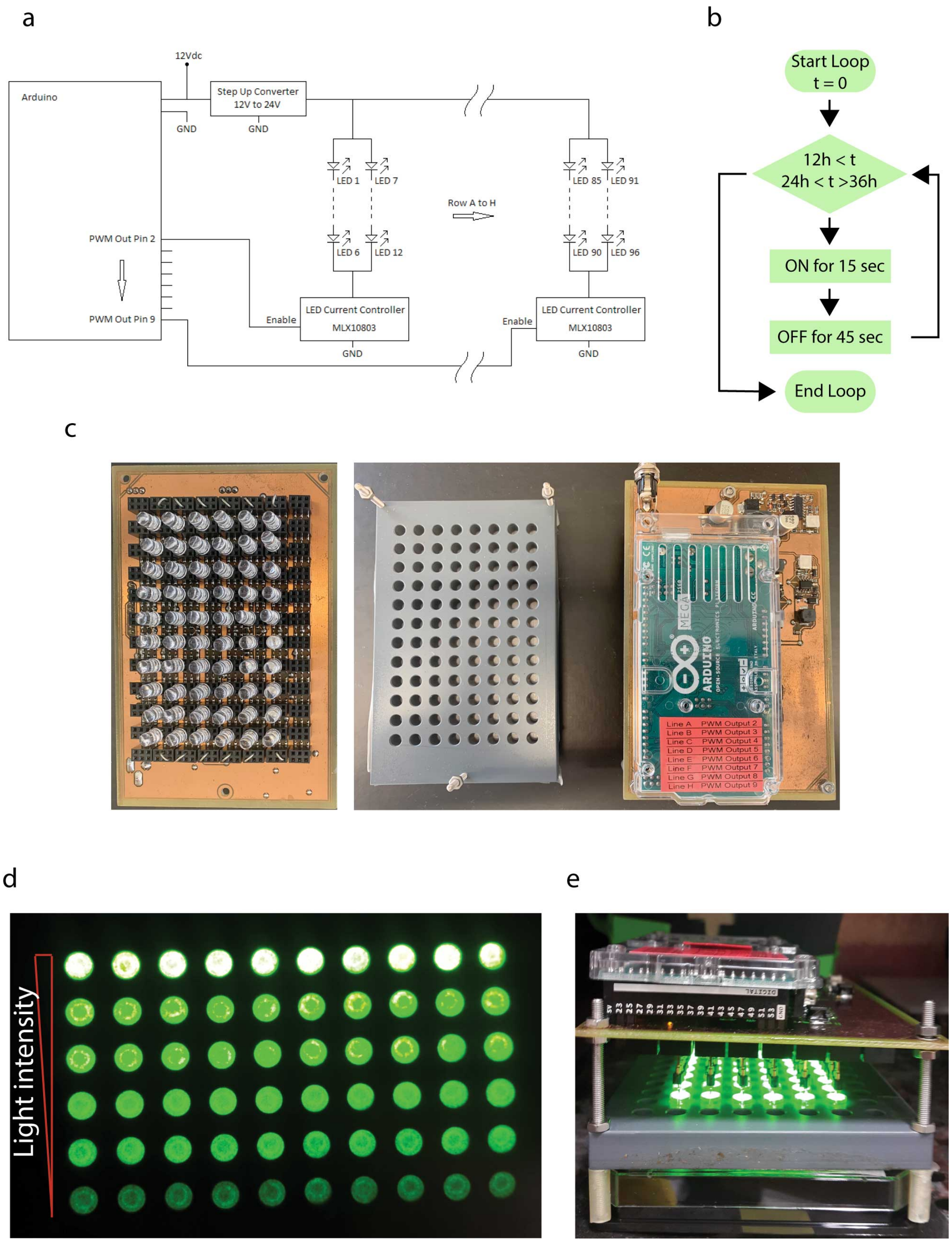

Supplementary Figure 9 | Construction of a multiwell light-induction platform for 96-well cell culture plate. **a**, Circuit diagram of the LED array. **b**, Flow-chart of the program running on the Arduino^{TM}. Pulses of e.g., 15 seconds ON and 45 seconds OFF were applied for 12 h each day. **c**, View of disassembled parts. Left: bottom view showing LED holders; middle: fitting spacer to ensure correct orientation of LEDs; right: top view of the Arduino^{TM} controller. **d**, Image of an operating LED array programmed with a light intensity gradient. **e**, Operating the LED array on a 96-well plate inside a mammalian cell culture incubator.

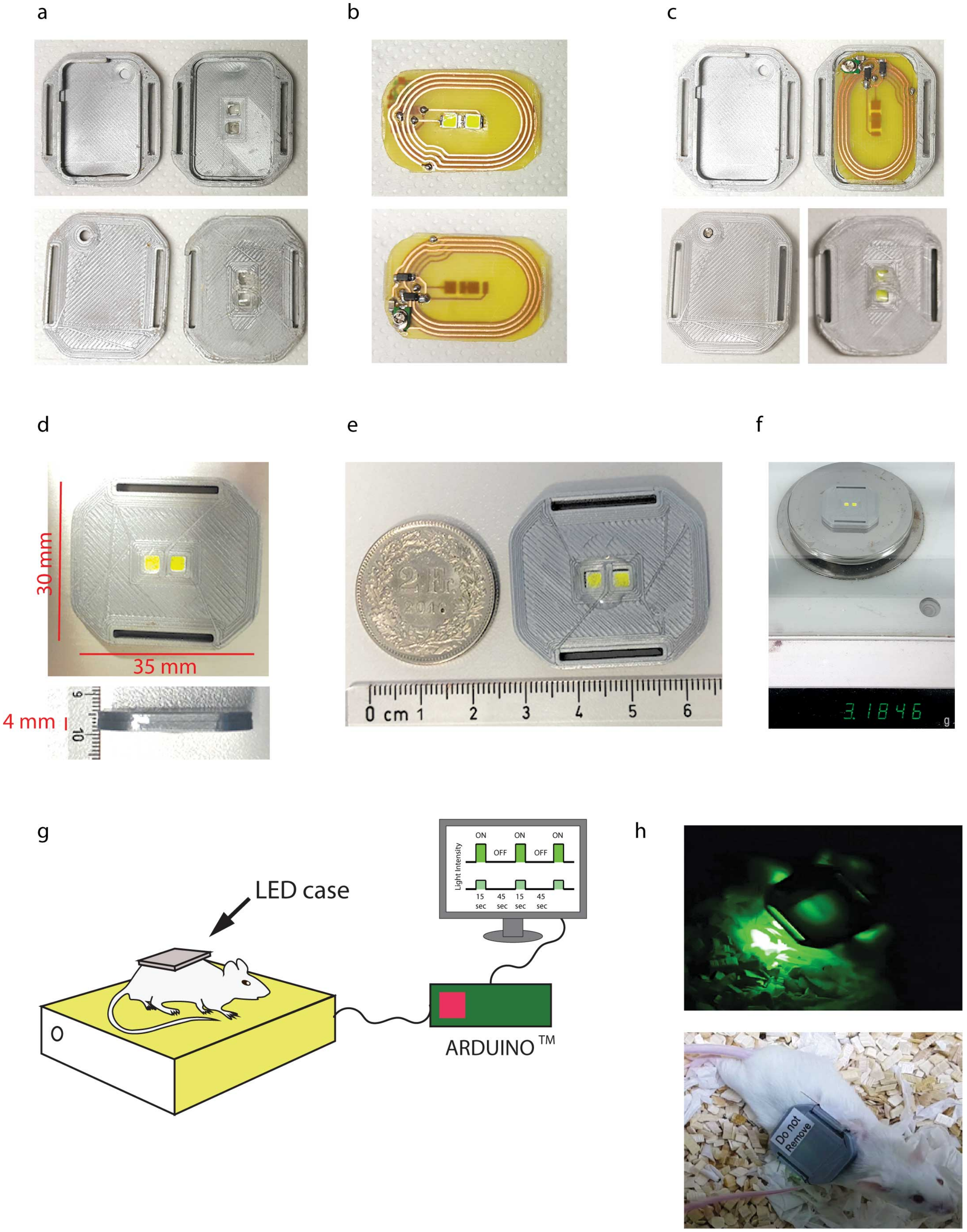

Supplementary Figure 10 | Design of the customized and programmable smart-watch-mimicking LED patch. The LED case is a 3D printed plastic frame containing a printed circuit

board (PCB) to harvest the energy from a magnetic field to drive two LEDs. **a**, A top view of the plastic case carrying the PCB, from inside (top) and outside (down). **b**, Top view of the PCB harboring green LEDs from the front (top) and back (down). **c**, The plastic case equipped with the PCB in an open (top) or closed and finalized (down) format. **d-f**, A functional smart-watch-mimicking LED patch; **d**, Dimensions, **e**, LED patch dimensions, with a coin for reference, and **f**, weight of the complete LED patch. **g**, The LED patch is stitched face down onto the mouse skin. A magnetic field generator provides wireless energy transmission to power the LEDs. The field generator is also connected to Arduino^{TM}, allowing control of light intensity and illumination time. **h**, working LED patch on a mouse in the light and in the dark.

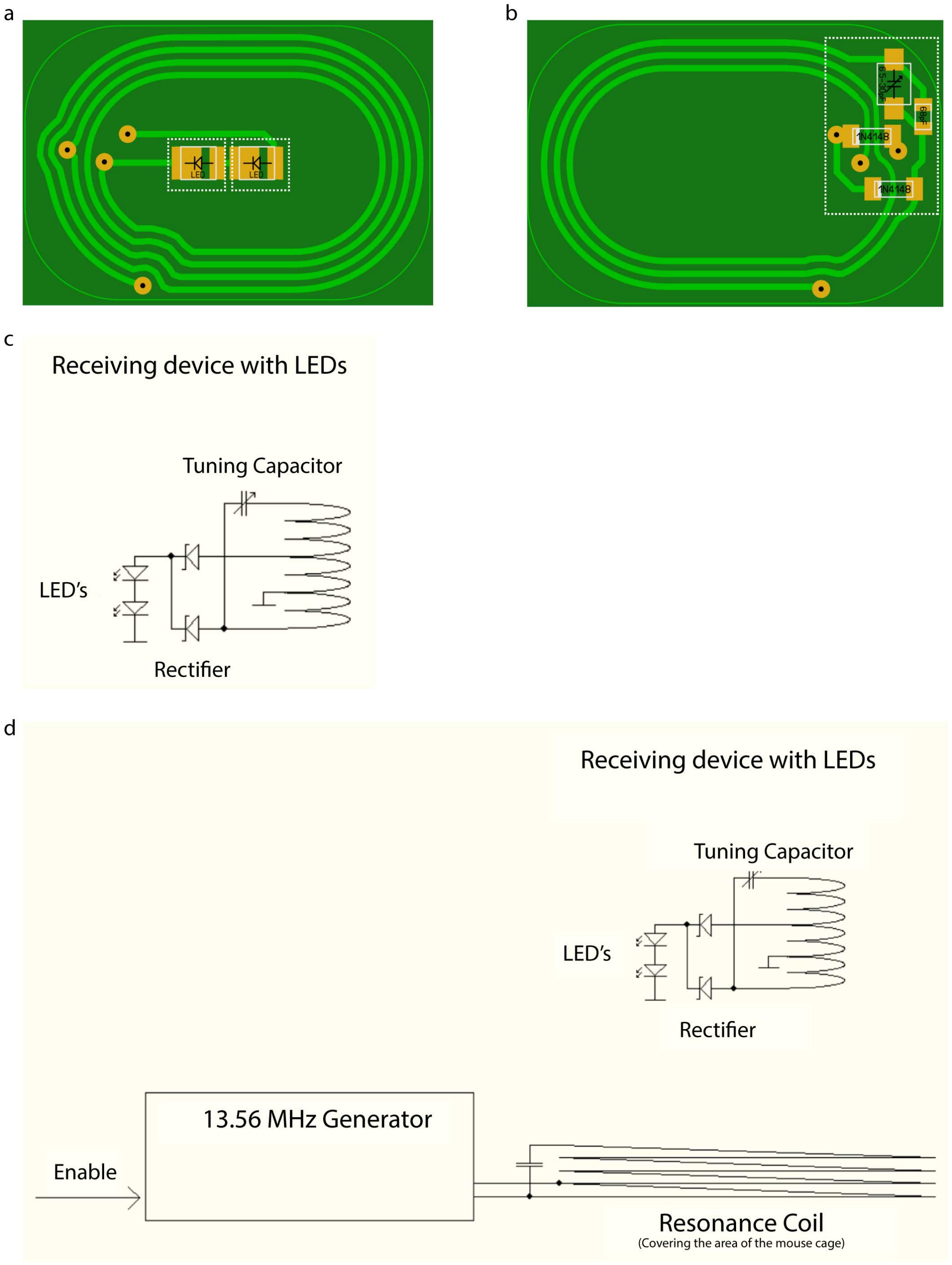

Supplementary Figure 11 | Electronic circuits of the LED patch and the field generator. a-b, Schematic view of the smart-watch-mimicking PBC from the top (**a**) and bottom (**b**). Conductive paths are marked with lighter green and orange rectangles represent connectors. White dashed frames in (a) and (b) indicate LEDs and the energy-harvesting circuit, respectively. **c**) Electronic circuit of the PBC architecture. **d**) Schematic circuit diagram of the magnetic field connected wirelessly to the LED patch.

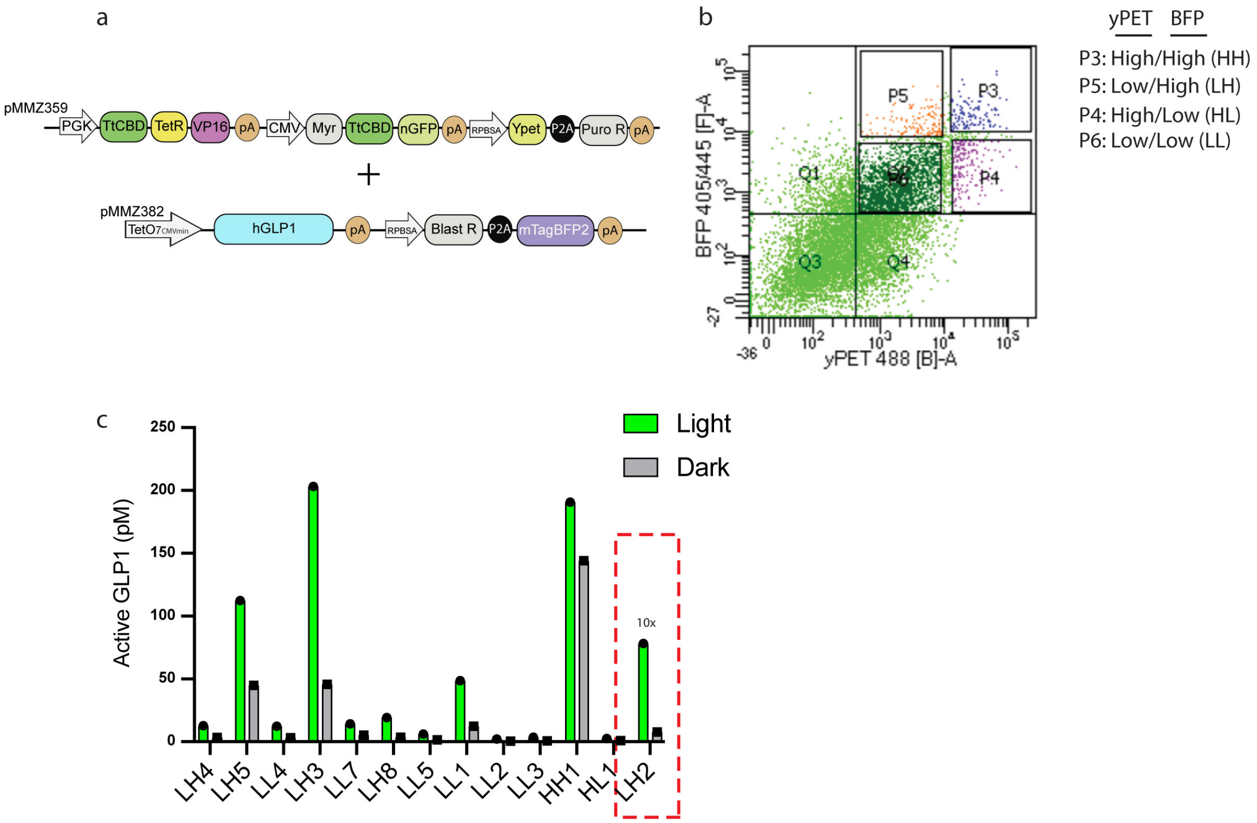

Supplementary Figure 12 | Generation of Glow Control$_{\text{-hGLP1}}$ stable cell line. a, HEK293 cells were co-transfected with green-light-responsive module (pMMZ359; P_{PGK}-TtCBD-TetR-VP16-pA_P_{CMV}-Myr-TtCBD-nGFP-pA_P_{RPBA}-Ypet-p2a-Puro-pA) and a therapeutic response module (pMMZ382; $P_{TetO7\text{-}CMVmin}$-hGLP1-pA_P_{RPBA}-BFP-p2a-BlastR-pA as well as pTS395 (P_{hCMV}-SB-pA) to generate a polyclonal cell population. **b**, The polyclonal cell population was sorted by FACS according to fluorescent protein intensity levels (yPET and BFP) and divided into four subpopulations (HH, HL, LH and LL). Each sorted cell was placed in a single well of a 96-well plate and was grown in selection media containing puromycin and blasticidin. **c**, Functional screening was performed by measuring hGLP1 expression in supernatant of cultured cells after green-light illumination for 48 h (545 nm, 12 h/day, 15 sec ON/45 sec OFF, and 88 µW/cm^2 cells). Control cells were kept in the dark. The red dashed frame indicates the selected best performer; 10x above the left bar indicate a 10-fold change of hGLP1 expression versus the adjacent dark control. Source data are provided as a Source Data file.

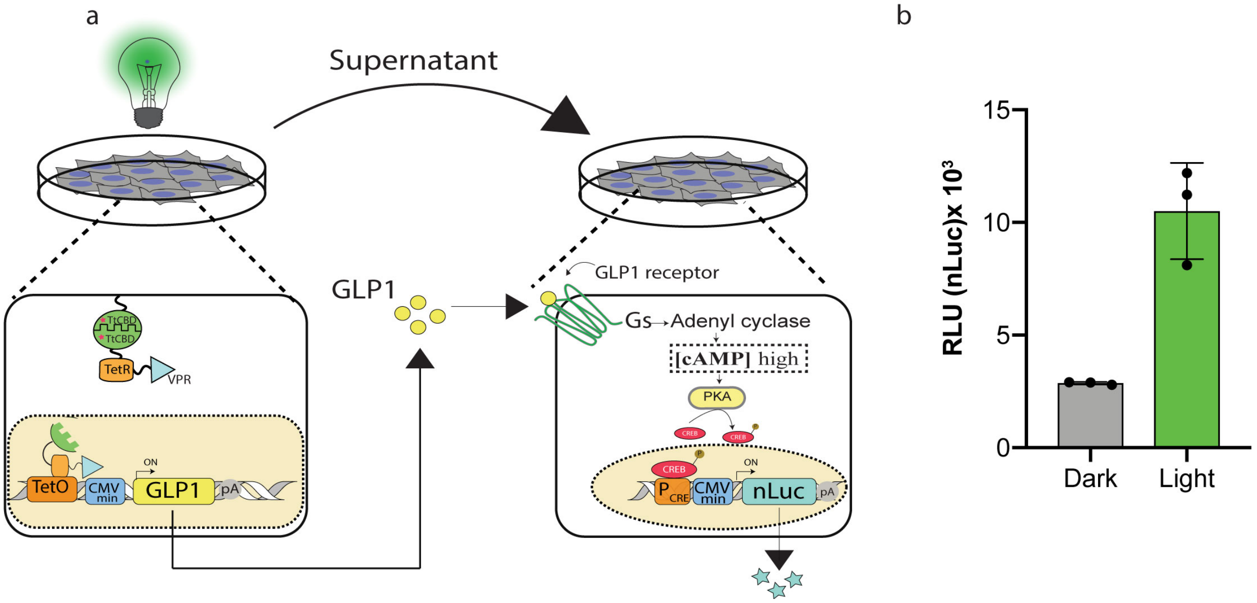

Supplementary Figure 13 | *In vitro* functional assessment of hGLP1 produced by Glow Control$_{\text{-hGLP1}}$ stable cell line. a, Schematic representation of green-light-triggered hGLP1 production in Glow Control$_{\text{-hGLP1-LH2}}$ cells (left side) which can induce NanoLuc expression in the GLP1 reporter cells (right side). Pulsated green light irradiation (545 nm, 12 h/day, 15 sec ON/45 sec OFF, and 88 $\mu W/cm^2$ cells) induced expression of hGLP1 and SEAP in the illuminated Glow Control$_{\text{-hGLP1-LH2}}$ stable cell line for 48 h (related to Fig. 4E). Supernatant of the illuminated cells was used to activate NanoLuc expression in HEK293T cells transfected with pGLP1R (P_{hCMV}-GLP1R-pA) and pTS309 (P_{CRE}-nLuc-pA). **b**, After incubation for 24 h, NanoLuc expression levels were quantified in the supernatant of cultured cells. Bars represent the mean ± s.d. (n = 3 independent experiments). Source data are provided as a Source Data file.

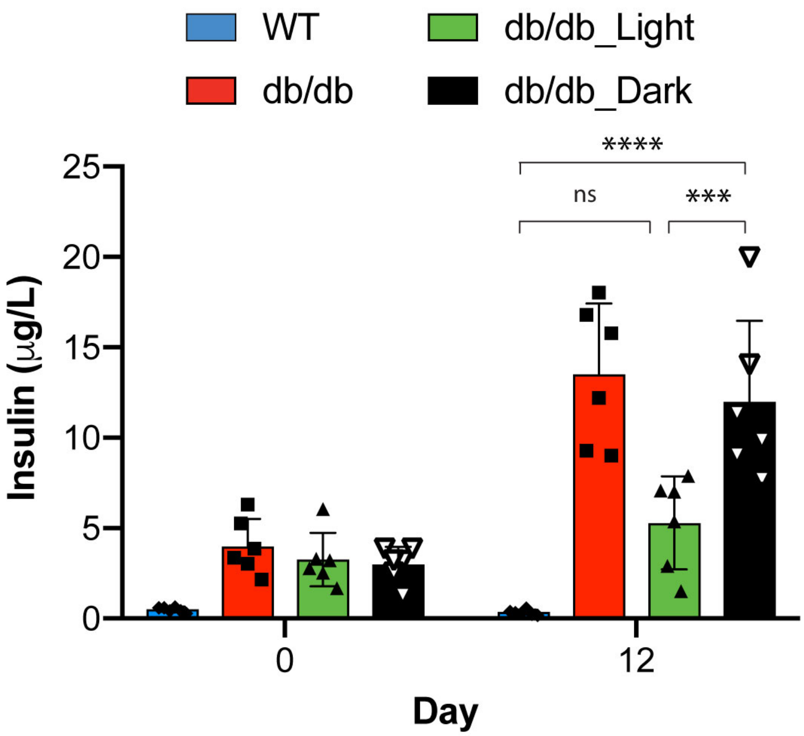

Supplementary Figure 14 | Characterization of insulin level upon green light activation of implanted Glow Control cells *in vivo*. Effect of hGLP1 expression on insulin levels in T2D mice after illumination with or without green light for 12 days (related to Fig. 4h). Bars represent the mean ± s.d. (n = 6). Statistical significance between $Lpr^{db/db}$ mice of the indicated groups on day 12 were calculated using a one-way ANOVA. ns, not significant, ***, p<0.001; ****, p<0.0001. Source data are provided as a Source Data file.

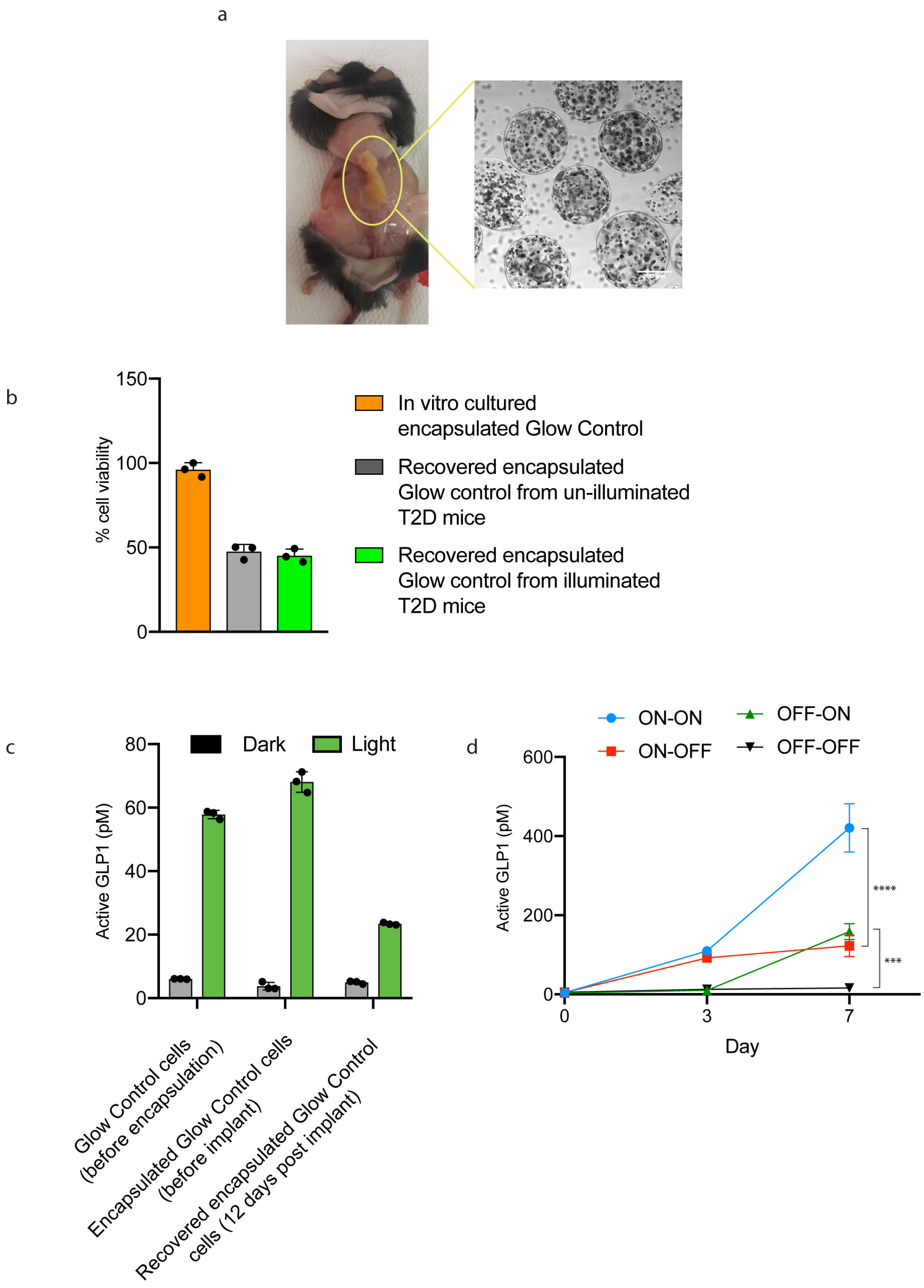

Supplementary Figure 15 | Functionality of the encapsulated Glow Control cells. a, Glow Control-hGLP1 cells encapsulated in alginate-poly(L-lysine)-alginate microcapsules were recovered from the dorsal subcutaneous space of sacrificed $Lpr^{db/db}$ mice 12 days after transplantation (left side). Encapsulated cells were washed with DMEM and imaged with a bright-field microscope (right side). Scale bar is 200 µm. A representative image of three replicates is shown. **b**, Viability of microencapsulated Glow Control-hGLP1 cells. 200 recovered capsules from illuminated and

un-illuminated $Lpr^{db/db}$ mice 12 days after transplantation, together with capsules that had been kept in an incubator for 12 days, were subjected to MTT assay (n = 3), **c**, In vitro quantitative evaluation of Glow $Control_{-hGLP1-LH2}$ before encapsulation, after encapsulation and after recovery on the 12th day after transplantation. Cells were either illuminated with pulsed green light (545 nm, 12 h/day, 15 sec ON/45 sec OFF, and 88 $\mu W/cm^2$ cells) for 48 h or kept in the dark. Bars represent the mean ± s.d. (n = 3 independent experiments). **d,** *In vitro* reversibility of Glow $Control_{-hGLP1}$ cells (related to Figure 4j) in the presence (ON) or absence (OFF) of pulsed green light (545 nm, 12 h/day, 30 sec ON/30 sec OFF, 150 $\mu W/cm^2$). Values are mean ± s.d. for n = 3 cell culture experiments. Statistical significance was calculated using a two-way ANOVA. ***, $p<0.001$; ****, $p<0.0001$. Source data are provided as a Source Data file.

Supplementary Table 1. Plasmids used and designed in this study.

Plasmid	Description and cloning strategy	Reference
**pMMZ260**	P_{CMV}-TtCBD-pA<br>Mammalian expression vector encoding the TtCBD. TtCBD was amplified with oMMZ104 and oMMZ105 from OptoOFF-TtCBD-mFGFR1_624[1] (addgene ID: 105826) and cloned into BB3-P_{CMV}-SEAP-PA cloning vector restricted with EcoRI/HindIII.	This work
**pMMZ261**	P_{CMV}-G_4S-TtCBD-pA<br>Mammalian expression vector encoding the TtCBD containing GGGGS linker in N-terminus. G_4S -TtCBD was amplified with oMMZ106 and oMMZ105 from OptoOFF-TtCBD-mFGFR1_624 (addgene ID: 105826) and cloned into BB3-Ph_{CMV}-SEAP-PA cloning vector restricted with EcoRI/HindIII.	This work
**pMMZ262**	P_{CMV}-TtCBD-G_4S-pA<br>Mammalian expression vector encoding the TtCBD containing GGGGS linker in C-terminus. TtCBD-G_4S was amplified with oMMZ104 and oMMZ107 from OptoOFF-TtCBD-mFGFR1_624 (addgene ID: 105826) and cloned into BB3-P_{CMV}-SEAP-PA cloning vector restricted with EcoRI/HindIII.	This work
**pMMZ295**	P_{SV40}-TtCBD-G_4S-VPR-pA<br>Mammalian expression vector encoding the TtCBD-G_4S-VPR.<br>VPR was provided by SpeI/HindIII digestion of pHV015 and cloned into pMMZ262 restricted with NheI/HindIII.	This work
**pMMZ271**	P_{SV40}-TtCBD-G_4S-TetR-pA<br>Mammalian expression vector encoding the TtCBD-G_4S-VPR.<br>TetR was provided by SpeI/HindIII digestion of pTS1252 and cloned into pMMZ262 restricted with NheI/HindIII.	This work
**pTS1017**	P_{TetO7}-$P_{hCMVmin}$-SEAP-pA<br>Mammalian reporter plasmid containing TetR binding sites upstream of a minimal promoter driving SEAP expression. $TetO_7$ was amplified with oTS733 and oTS734 from pDA326[2].	This work
**pMMZ269**	P_{SV40}-Myr(SP)-TtCBD-pA<br>Mammalian expression vector encoding Myr-TtCBD.<br>TtCBD was provided by SpeI/HindIII digestion of pMMZ260 and cloned into pVH017 plasmid restricted with NheI/HindIII.	This work
**pMMZ273**	P_{SV40}- TtCBD-G_4S-TetR-VPR-pA<br>Mammalian expression vector encoding the TtCBD-TetR-VPR.<br>VPR was provided by SpeI/HindIII digestion of pVH015 and cloned into pMMZ271 plasmid restricted with NheI/HindIII.	This work
**pMMZ351**	P_{SV40}- Myr-TtCBD-eGFP-pA<br>Mammalian expression vector encoding the Myr-TtCBD-eGFP.<br>eGFP was provided by SpeI/HindIII digestion of pFOX12 and cloned into pMMZ269 plasmid restricted with NheI/HindIII.	This work
**pMMZ272**	P_{SV40}-TtCBD-G_4S-TetR-G_4S-VP16-pA<br>Mammalian expression vector encoding the TtCBD-TetR-VP16.<br>VP16 was provided by SpeI/HindIII digestion of pTS1251 and cloned into pMMZ271 plasmid restricted with NheI/HindIII.	This work
**pMMZ283**	P_{SV40}-Myr-TtCBD-pGFP-pA<br>Mammalian expression vector encoding the Myr-TtCBD-pGFP.<br>pGFP was provided by SpeI/HindIII digestion of pLeo1087 and cloned into pMMZ269 plasmid restricted with NheI/HindIII.	This work
**pMMZ284**	P_{SV40}-Myr-TtCBD-nGFP-pA<br>Mammalian expression vector encoding the Myr-TtCBD-nGFP.<br>nGFP was provided by SpeI/HindIII digestion of pLeo1088 and cloned into pMMZ269 plasmid restricted with NheI/HindIII.	This work
**pMMZ290**	P_{SV40}-pGFP-G_4S-TtCBD- G_4S-TetR-G_4S-VP16-pA	This work

	Mammalian expression vector encoding the pGFP-TtCBD-TetR-VP16.<br>TtCBD-TetR-VP16 was provided by SpeI/HindIII digestion of pMMZ287 and cloned into pLeo1087 plasmid restricted with NheI/HindIII.	
**pMMZ291**	P_{SV40}-nGFP-G$_4$S-TtCBD-G$_4$S-TetR-G$_4$S-VP16-pA<br>Mammalian expression vector encoding the pGFP-TtCBD-TetR-VP16.<br>TtCBD-TetR-VP16 was provided by SpeI/HindIII digestion of pMMZ287 and cloned into pLeo1088 plasmid restricted with NheI/HindIII.	This work
**pMMZ287**	P_{SV40}- G$_4$S-TtCBD-G$_4$S-TetR-G$_4$S-VP16-pA<br>Mammalian expression vector encoding the G4S-TtCBD-TetR-VP16.<br>TtCBD-TetR-VP16 was provided by SpeI/HindIII digestion of pMMZ272 and cloned into pMSBB33 plasmid restricted with NheI/HindIII.	This work
**pMMZ309**	P_{SV40}-GFP-G$_4$S-TtCBD-G$_4$S-TetR-VP16-pA<br>Mammalian expression vector encoding the GFP-TtCBD-TetR-VP16.<br>G$_4$S-TtCBD-TetR-G$_4$S-VP16 was provided by SpeI/HindIII digestion of pMMZ287 and cloned into pFOX12 plasmid restricted with NheI/HindIII.	This work
**pMMZ304**	P_{SV40}-TtCBD-G$_4$S-TetR-VP64-pA<br>Mammalian expression vector encoding the TtCBD-TetR-VP64.<br>VP64 was provided by SpeI/HindIII digestion of pGM45 and cloned into pMMZ271 plasmid restricted with NheI/HindIII.	This work
**pLeo1403**	P_{TetO7}-$P_{hCMVmin}$-nLuc-Fc-pA<br>Mammalian reporter plasmid containing TetR binding sites upstream of a minimal promoter driving secreted nLuc expression.	This work
**pQP-T2A**	P_{CMV}- BphP1-VP16-T2A-NLS-GAL4-QPAS1-pA<br>Mammalian expression vector encoding the near-infrared light responsive elements (BphP1 and QPAS1) fused to VP16 and GAL4 (addgene ID: 102583).	Redchuk et al.[3]
**pSP30**	P_{UAS5}-SEAP-pA<br>Mammalian reporter plasmid containing UAS binding sites upstream of a minimal promoter driving SEAP expression.	Saxena et al.[4]
**pMMZ355**	P_{PGK}-TtCBD-G$_4$S-TetR-G$_4$S-VP16-pA<br>Mammalian expression vector encoding the TtCBD-TetR-VP16.<br>TtCBD-TetR-VP16 was restricted with EcoRI/HindIII from pMMZ272 and ligated into the corresponding sites (EcoRI/HindIII) of pMM328.	This work
**pMMZ354**	P_{CMV}-Myr-TtCBD-nGFP-pA<br>Mammalian expression vector encoding the Myr-TtCBD-nGFP.<br>Myr-TtCBD-nGFP was restricted with EcoRI/HindIII from pMMZ284 and ligated into the corresponding sites (EcoRI/HindIII) of pTS1022.	This work
**pMMZ410**	P_{CMV}-Myr-nGFP-G$_4$S-TtCBD-pA<br>Mammalian expression vector encoding the Myr-nGFP-TtCBD.<br>Myr-nGFP was restricted with SacI/NheI from pMMZ408 and ligated into pMMZ261 plasmid digested with (SacI/SpeI).	This work
**pMMZ359**	P_{PGK}-TtCBD-G$_4$S-TetR-G$_4$S-VP16-pA_ P_{CMV}-Myr-TtCBD-nGFP-pA_P_{RBPA}-Ypet-p2a-Puro-pA<br>ITR-containing vector for SB100X[5]-specific transposon mediated stable genomic integrations, containing a constitutive BlastR and BFP expression unit, and two constitutive expression units for green responsive module (ITR-P_{PGK}-TtCBD-G$_4$S-TetR-G$_4$S-VP16-pA_ P_{CMV}-Myr-TtCBD-nGFP-pA_P_{RBPA}-Ypet-p2a-Puro-pA-ITR).<br>pMMZ355 (P_{PGK}-TtCBD-G$_4$S-TetR-G$_4$S-VP16-pA) was restricted with MluI/HindIII and ligated into the corresponding sites (MluI/HindIII) of pTS1023 to generate pMMZ358. Next, pMMZ354 digested with MluI/HindIII and cloned into pMMZ358 restricted with BsaI.	This work
**pMMZ380**	P_{TetO7}-$P_{hCMVmin}$-hGLP1-2A-SEAP-pA<br>hGLP1-2A-SEAP was amplified with oMMZ155/oMMZ115 from pHY102[6] plasmid. PCR fragment was digested with EcoRI/HindIII and ligated into the corresponding sites (EcoRI/HindIII) of pTS1017.	This work
**pMMZ382**	$P_{TetO7-hCMVmin}$-hGLP1 -pA_ P_{RBPA}-Blasticidin-p2a-BFP-pA	This work

	ITR-containing vector for SB100X-specific transposon mediated stable genomic integrations, containing a constitutive BlastR and BFP expression unit, and a TetO7 reporter driving expression of a modified version of hGLP1 with enhanced stability (ITR- P_{TetO7}-$P_{hCMVmin}$-hGLP1-pA_ P_{RBPA}-Blasticidin-p2a-BFP-pA-ITR). pMMZ380 was restricted with MluI/HindIII and ligated into the corresponding sites (MluI/HindIII) of pTS1024.	
**pMMZ429**	P_{SV40}- TtCBD-G4S-G4S-TetR-VPR-mCherry-pA<br>Mammalian expression vector encoding the TtCBD-TetR-VPR-mCherry. mCherry was restricted with SpeI/HindIII from BB3-mCherry and ligated into pMMZ273 plasmid digested with (NheI/HindIII).	This work
**pVH015**	P_{hCMV}-VPR-pA<br>Mammalian expression plasmid encoding VPR (Vp64-p65-Rta) transactivator domain (NLS between VP16 and p65).	Unpublished
**pTS1252**	P_{hCMV}-TetR-pA<br>Mammalian expression plasmid encoding TetR domain.	This work
**pVH017**	P_{hCMV}-Myristoylation (SS)-pA<br>Mammalian expression plasmid encoding Myristoylation sequence (MGCINSKRKD).	Unpublished
**pFOX12**	P_{hCMV}-eGFP-pA<br>Mammalian expression plasmid encoding eGFP.	Unpublished
**pTS1251**	P_{hCMV}-VP16-pA<br>Mammalian expression plasmid encoding VP16.	This work
**pGM45**	P_{hCMV}-VP64-pA<br>Mammalian expression plasmid encoding VP64.	Unpublished
**pTS1022**	P_{hCMV}-SEAP-pA<br>Mammalian expression plasmid with a CMV promoter driving expression of SEAP in BB3 backbone.	This work
**pMM328**	P_{PGK}-SEAP-pA<br>Mammalian expression plasmid with a PGK promoter driving expression of SEAP in BB3 backbone.	Müller et al.[7]
**pTS1016**	P_{SV40}-SEAP-pA<br>Mammalian expression plasmid with a SV40 promoter driving expression of SEAP in BB3 backbone.	This work
**pTS1023**	ITR-containing vector for SB100X-specific transposon mediated stable genomic integrations, containing a constitutive Ypet and PuroR expression unit.	This work
**pTS1024**	ITR-containing vector for SB100X-specific transposon mediated stable genomic integrations, containing a constitutive BlastR and BFP expression unit.	This work
**pTS395**	P_{hCMV}-SB-pA<br>Mammalian expression plasmid for SB expression.	This work
**pDF145**	Bacterial RNA production vector without mammalian promoter activity (P_{T7}-SpAH-Env140ac).	Fuchs et al.[8]
**pTS309**	P_{CRE}-Igk-nLuc-pA<br>Mammalian reporter plasmid for cAMP-induced expression of NanoLuc.	This work
**pGLP1R**	P_{CMV}-GLP1R-pA<br>Mammalian expression plasmid encoding Glucagon-like peptide-1 receptor (GLP1R).	Xue et al.[9]
**pMMZ516**	P_{CMV}-HA-TtCBD-G_4S-TetR-G_4S-VP16-pA<br>Mammalian expression vector encoding the HA-TtCBD-TetR-VP16.	This work
**pMMZ518**	P_{CMV}-UbK-HA-TtCBD-G_4S-TetR-G_4S-VP16-pA<br>Mammalian expression vector encoding the UbK-HA-TtCBD-TetR-VP16.	This work

Supplementary Table 2. Oligonucleotides utilized for PCR in this study.

	Name	sequence
**1**	oMMZ104	AGCGGAATTCACCATGACTAGTcccgaggacctcggcaccggac
**2**	oMMZ105	CTCAAAGCTTTCTAGACACCGGTGGATCCGCTAGCgattgcttctttctctggtcctcttg
**3**	oMMZ106	AGCGGAATTCACCATGACTAGTGGAGGTGGAGGTAGTcccgaggacctcggcaccggac
**4**	oMMZ107	CTCAAAGCTTTCTAGACACCGGTGGATCCGCTAGCACTACCTCCACCTCCgattgcttctttctctggtcctcttg
**5**	oTS733	CTGAACGCGTCCGTACACGCCTAAAGCATATACGTTC
**6**	oTS734	CCTCGACATACTCGAGTTTACTCCCTATC
**7**	oMMZ155	atcg*GAATTC*ATGAAGATCATCCTGTGGCTG
**8**	oMMZ115	CTCAAAGCTTTCTAGACACCGGTGGATCCGCTAGCTGTCTGCTCGAAGCGG
**9**	oMMZ156	atcgGAATTCATGCTGCTGCTGCTGCTGCTG
**10**	oMMZ149	agtcAAGCTTTCAGTTGCAGTAGTTCTCCAGTTGGTAG

Supplementary Note 1. Arduino control script for the custom-designed photostimulation device.

```
int ledRowA = 2;    // LED connected to digital pin 2
int ledRowB = 3;    // LED connected to digital pin 3
int ledRowC = 4;    // LED connected to digital pin 4
int ledRowD = 5;    // LED connected to digital pin 5
int ledRowE = 6;    // LED connected to digital pin 6
int ledRowF = 7;    // LED connected to digital pin 7
int ledRowG = 8;    // LED connected to digital pin 8
int ledRowH = 9;    // LED connected to digital pin 9

long wait_time1 = 15000; // time LED is ON in ms
long wait_time2 = 45000; // time LED is OFF in ms
long time_ON_cycle = 60; // time of pulse-cycles in s
long time_OFF_cycle = 0; // time of total darkness in s
long total_induction_time = 720; // total time of experiment in min

unsigned long StartTime1 = millis();
unsigned long CurrentTime = millis();
unsigned long ElapsedTime1 = CurrentTime - StartTime1;

unsigned long StartTime2 = millis();
unsigned long ElapsedTime2 = CurrentTime - StartTime2;


void setup() {
  // nothing happens in setup
}

void loop() {
   // sets the value (range from 0 to 255) on the ledPin specified above
   while (ElapsedTime1<(total_induction_time*60000) ) {

   StartTime2 = millis();
   ElapsedTime2 = CurrentTime - StartTime2;
     while (ElapsedTime2<(time_ON_cycle*1000)) {
       // ON
       analogWrite(ledRowA, 100);
       analogWrite(ledRowB, 100);
       analogWrite(ledRowC, 100);
       analogWrite(ledRowD, 100);
       analogWrite(ledRowE, 100);
       analogWrite(ledRowF, 100);
```

```
        analogWrite(ledRowG, 100);
        analogWrite(ledRowH, 100);
        // wait for x milliseconds
        delay(wait_time1);

        // OFF
        analogWrite(ledRowA, 0);
        analogWrite(ledRowB, 0);
        analogWrite(ledRowC, 0);
        analogWrite(ledRowD, 0);
        analogWrite(ledRowE, 0);
        analogWrite(ledRowF, 0);
        analogWrite(ledRowG, 0);
        analogWrite(ledRowH, 0);
        // wait for x milliseconds
        delay(wait_time2);
        CurrentTime = millis();
        ElapsedTime2 = CurrentTime - StartTime2;
      }

    // pause in-between cycles
    delay(time_OFF_cycle*1000);
    CurrentTime = millis();
    ElapsedTime1 = CurrentTime - StartTime1;
    }
}
```

Abbreviations.
TtCBD: Cobalamin-binding domain from *Thermus thermophilus*; **P_{hCMV}**, human cytomegalovirus immediate early promoter; **$P_{hCMVmin}$**, minimal human cytomegalovirus immediate early promoter, **P_{SV40}**, simian virus 40 promoter; **P_{PGK}**, simian virus 40 promoter; **P_{RPBSA},** constitutive synthetic mammalian promoter; **SEAP**, human placental secreted alkaline phosphatase; **NanoLuc (nLuc)**, *Oplophorus gracilirostris* luciferase; **shGLP1**, short human glucagon-like peptide 1; **mINS**, mouse insulin; **YPet,** yellow fluorescent protein variant; **BFP**, blue fluorescent protein; **GFP**, green fluorescent protein; **BlastR**, blasticidin resistance gene; **PuroR,** puromycin resistance gene; **ITR**, inverted terminal repeats of SB100X; **SB100X**, optimized Sleeping Beauty transposase; **P2A,** 2A peptide, **pA,** polyadenylation signal, **SP**, secretory signal peptide; **Fc**, Fc fragment of murine IgG antibody; **MCS**, multiple cloning site; **NLS**, nuclear localization signal; **TetO**, TetR-binding operator sequence; **p65**, human transcription factor p65; **pA**, polyadenylation signal; **PCR**, polymerase chain reaction; **G_4S**, Gly-Gly-Gly-Gly-Ser; **Myr**, Myristoylation signal; **VPR,** Vp16-p65-Rta transactivator; **nGFP**, negatively supercharged GFP; **pGFP**, positively supercharged GFP; **BphP1**, bacterial phytochrome P1; **QPAS1**, engineered protein partner of BphP1; **UAS,** upstream activation sequence, PFA, paraformaldehyde.

References
1. Kainrath, S., Stadler, M., Reichhart, E., Distel, M. & Janovjak, H. Green-light-induced inactivation of receptor signaling using cobalamin-binding domains. *Angew. Chemie - Int. Ed.* **56**, 4608–4611 (2017).
2. Ausländer, D. *et al.* Programmable full-adder computations in communicating three-dimensional cell cultures. *Nat. Methods* **15**, 57–60 (2018).
3. Redchuk, T. A., Omelina, E. S., Chernov, K. G. & Verkhusha, V. V. Near-infrared optogenetic pair for protein regulation and spectral multiplexing. *Nat. Chem. Biol.* **13**, 633–639 (2017).
4. Saxena, P., Hamri, G. C. El, Folcher, M., Zulewski, H. & Fussenegger, M. Synthetic gene network restoring endogenous pituitary-thyroid feedback control in experimental Graves' disease. *Proc. Natl. Acad. Sci. U. S. A.* **113**, 1244–1249 (2016).
5. Mátés, L. *et al.* Molecular evolution of a novel hyperactive Sleeping Beauty transposase enables robust stable gene transfer in vertebrates. *Nat. Genet.* **41**, 753–761 (2009).
6. Ye, H., Baba, M. D. El, Peng, R. W. & Fussenegger, M. A synthetic optogenetic transcription device enhances blood-glucose homeostasis in mice. *Science (80-.).* **332**, 1565–1568 (2011).

7. Müller, M. *et al.* Designed cell consortia as fragrance-programmable analog-to-digital converters. *Nat. Chem. Biol.* **13**, 309–316 (2017).
8. Ausï Ander, S., Fuchs, D., Urlemann, S. H. ¨, Ausï Ander, D. & Fussenegger, M. Engineering a ribozyme cleavage-induced split fluorescent aptamer complementation assay. *Nucleic Acids Res.* **44**, 94 (2016).
9. Xue, S. *et al.* A Synthetic-Biology-Inspired Therapeutic Strategy for Targeting and Treating Hepatogenous Diabetes. *Mol. Ther.* **25**, 443–455 (2017).
