# Supplementary material for: Smart-watch-programmed green-light-operated percutaneous control of therapeutic transgenes
Source: Nat Commun. 2021 Jun 7;12:3388. doi: 10.1038/s41467-021-23572-4 (PMC8184832; doi:10.1038/s41467-021-23572-4)
Supplement: Supplementary file 3 — Reporting Summary [file 41467_2021_23572_MOESM3_ESM.pdf]

nature research

Corresponding author(s): Prof. Dr. Martin Fussenegger

Last updated by author(s): Apr 22, 2021

Reporting Summary

Nature Research wishes to improve the reproducibility of the work that we publish. This form provides structure for consistency and transparency in reporting. For further information on Nature Research policies, see our Editorial Policies and the Editorial Policy Checklist.

Statistics

For all statistical analyses, confirm that the following items are present in the figure legend, table legend, main text, or Methods section.

n/a	Confirmed	
☐	☒	The exact sample size (*n*) for each experimental group/condition, given as a discrete number and unit of measurement
☐	☒	A statement on whether measurements were taken from distinct samples or whether the same sample was measured repeatedly
☐	☒	The statistical test(s) used AND whether they are one- or two-sided<br>*Only common tests should be described solely by name; describe more complex techniques in the Methods section.*
☒	☐	A description of all covariates tested
☐	☒	A description of any assumptions or corrections, such as tests of normality and adjustment for multiple comparisons
☐	☒	A full description of the statistical parameters including central tendency (e.g. means) or other basic estimates (e.g. regression coefficient) AND variation (e.g. standard deviation) or associated estimates of uncertainty (e.g. confidence intervals)
☐	☒	For null hypothesis testing, the test statistic (e.g. *F*, *t*, *r*) with confidence intervals, effect sizes, degrees of freedom and *P* value noted<br>*Give P values as exact values whenever suitable.*
☒	☐	For Bayesian analysis, information on the choice of priors and Markov chain Monte Carlo settings
☒	☐	For hierarchical and complex designs, identification of the appropriate level for tests and full reporting of outcomes
☒	☐	Estimates of effect sizes (e.g. Cohen's *d*, Pearson's *r*), indicating how they were calculated

Our web collection on statistics for biologists contains articles on many of the points above.

Software and code

Policy information about availability of computer code

Data collection	Absorbance and luminescence data was collected using TECAN AG, Maennedorf, Switzerland.<br>Microscopic images were collected using Leica SP8 laser scanning confocal microscope.
Data analysis	GraphPad Prism 8, FlowJo 10.5, ImageJ 1.51 or Fiji 2

For manuscripts utilizing custom algorithms or software that are central to the research but not yet described in published literature, software must be made available to editors and reviewers. We strongly encourage code deposition in a community repository (e.g. GitHub). See the Nature Research guidelines for submitting code & software for further information.

Data

Policy information about availability of data

All manuscripts must include a data availability statement. This statement should provide the following information, where applicable:

- Accession codes, unique identifiers, or web links for publicly available datasets
- A list of figures that have associated raw data
- A description of any restrictions on data availability

The authors declare that all data supporting the findings of this study are available within the paper and its supplementary information files. Sequencing data of Glow Control plasmids have been deposited in GenBank under accession codes (pMMZ284; MW731449, pMMZ272; MW731450 and pTS1017; MT267334.1). All vector information is provided in Supplementary Table 1. Source data are provided with this paper. Requests for materials should be made to the corresponding author. All plasmids generated in this study are available upon request.

Field-specific reporting

Please select the one below that is the best fit for your research. If you are not sure, read the appropriate sections before making your selection.

☒ Life sciences ☐ Behavioural & social sciences ☐ Ecological, evolutionary & environmental sciences

For a reference copy of the document with all sections, see nature.com/documents/nr-reporting-summary-flat.pdf

Life sciences study design

All studies must disclose on these points even when the disclosure is negative.

Sample size	No statistical methods were used to predetermine sample size. Sample size was determined based on similar studies in our lab and other published studies in our field (Scheller L, Nat. Commun. 2020; Krawczyk K, Nat. Commun. 2020; Krawczyk K, Science. 2020). n=3 biologically independent samples were predicted to be sufficient for detecting statistically relevant differences between compared groups in cell culture experiments.
Data exclusions	All data was included.
Replication	Attempts at replication were successful. Replication times are detailed in each figure legend.
Randomization	For each mouse study, animals of the same genetic background were randomly allocated into different experimental groups. For cell culture experiments, no covariates based on sample allocations to experimental groups could be observed and no randomization was performed. all direct comparison of illuminated vs. un-illuminated were performed with cells transfected under the same conditions with the same transfection mixture.
Blinding	The investigators were not blinded to allocation during experiments and outcome assessment. Blinding was not possible as the same investigator processed the animal/cell culture experiments and analyzed the data.

Reporting for specific materials, systems and methods

We require information from authors about some types of materials, experimental systems and methods used in many studies. Here, indicate whether each material, system or method listed is relevant to your study. If you are not sure if a list item applies to your research, read the appropriate section before selecting a response.

Materials & experimental systems

n/a	Involved in the study	
☐	☒	Antibodies
☐	☒	Eukaryotic cell lines
☒	☐	Palaeontology and archaeology
☐	☒	Animals and other organisms
☒	☐	Human research participants
☒	☐	Clinical data
☒	☐	Dual use research of concern

Methods

n/a	Involved in the study	
☒	☐	ChIP-seq
☐	☒	Flow cytometry
☒	☐	MRI-based neuroimaging

Antibodies

Antibodies used	The anti-HA tag, is a monoclonal mouse antibody from Sigma Aldrich(H3663, diluted 1:10'000).<br>The anti-GAPDH, is a polyclonal Rabbit antibody from Abcam (ab9485, diluted 1:2500).<br>The alkaline phosphatase-coupled ECLTM anti-mouse from GE healthcare (NA931V, diluted 1:10'000) and alkaline phosphatase-coupled ECLTM anti-Rabbit IgGs from GE healthcare(NA934, diluted 1:10'000) were used as secondary antibodies.
Validation	We used a un-transfected cells as control to validate specificity of the antibodies. anti-HA and anti-GAPDH are validated for western blotting (e.g., Narayanan, S.R., J. Chromatogr., 1994; Pedone E, Nat. Commun., 2019).

Eukaryotic cell lines

Policy information about cell lines

Cell line source(s)	Human embryonic kidney cells (HEK293T, ATCC: CRL-3216), adipose tissue-derived human telomerase reverse transcriptase-immortalized human mesenchymal stem cells (hMSC-hTERT, ATCC: SCRC4000) and CV-1 (simian)-derived cells carrying SV40 (COS-7, ATCC: CRL1651)
Authentication	Cell were authenticated by ATCC. All the phenotype of cell lines was frequently checked and controlled by microscopy.

Mycoplasma contamination	HEK293T cells were tested for mycoplasma and confirmed as negative. Other cell types were not further tested for mycoplasma contamination. We also regularly check bacterial contaminations with all routinely used cell lines.
Commonly misidentified lines (See ICLAC register)	Cell lines used in the study are not listed in the database.

Animals and other organisms

Policy information about studies involving animals; ARRIVE guidelines recommended for reporting animal research

Laboratory animals	BKS-Leprdb/db/JOrlRj, RjOrl:SWISS (CD-1) and C57BL/6JRj. Mice were obtained from Janvier Labs (Saint-Bertheyin, France) and acclimatized for at least 1 week. Male and female mice aged 6-8 weeks were used.
Wild animals	The study did not involve wild animals.
Field-collected samples	The study did not involve field-collected samples.
Ethics oversight	All experiments involving animals were performed in accordance with the Swiss animal welfare legislation and approved by the veterinary office of the Canton Basel-Stadt (approval no. 2879/31996).

Note that full information on the approval of the study protocol must also be provided in the manuscript.

Flow Cytometry

Plots

Confirm that:

- [x] The axis labels state the marker and fluorochrome used (e.g. CD4-FITC).
- [x] The axis scales are clearly visible. Include numbers along axes only for bottom left plot of group (a 'group' is an analysis of identical markers).
- [ ] All plots are contour plots with outliers or pseudocolor plots.
- [x] A numerical value for number of cells or percentage (with statistics) is provided.

Methodology

Sample preparation	The sample preparation is described in details in the Methods section of the manuscript. Briefly, a polyclonal population of HEK293T cells were prepared in FACS sorting buffer (PBS with 0.2%FBS) and were filtrated before sorting via a 5 ml Polystyrene tube with Cell-Strainer Cap 35µm, Falcon #352235. Cells were kept on ice and were sorted into four different subpopulations according to their Ypet and BFP fluorescence intensities (High/High, High/Low, Low/High and Low/Low). Each individual sorted cell was grown in a well of a 96-well plate containing collection media (DMEM + 10% FBS+ 500 ug/ml Pen-Strep).
Instrument	Flow cytometry analysis was performed on a Fortessa flow cytometer (BD Biosciences).
Software	Flow cytometry data were analyzed with FlowJo 10.5 software.
Cell population abundance	Using fluorescent output, positive cells for Ypet and BFP signals were sorted into 96-well plate containing collection media (DMEM + 10% FBS+ 500 ug/ml Pen-Strep). 50-60% of sorted cells could grow and make monolayer in a well of 96-well plate. These monoclonal cell populations were screened for green-light-responsive GLP1 expression.
Gating strategy	A comprehensive report is attached in supplementary file. Gating for positive cells was performed based on wT cells expressing no fluorophore.

- [x] Tick this box to confirm that a figure exemplifying the gating strategy is provided in the Supplementary Information.
