# Supplementary material for: Smart-watch-programmed green-light-operated percutaneous control of therapeutic transgenes
Source: Nat Commun. 2021 Jun 7;12:3388. doi: 10.1038/s41467-021-23572-4 (PMC8184832; doi:10.1038/s41467-021-23572-4)
Supplement: Supplementary file 4 — Source Data [file 41467_2021_23572_MOESM4_ESM.zip › Data Source/FACS_report_related to Fig_SI12_.pdf]

BD FACSDiva 8.0.1

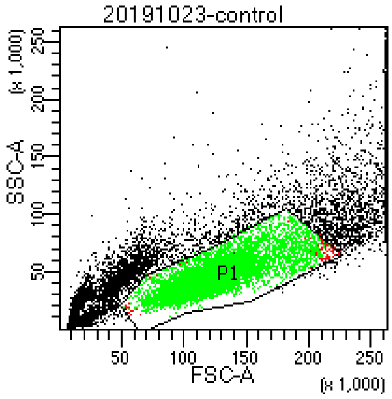

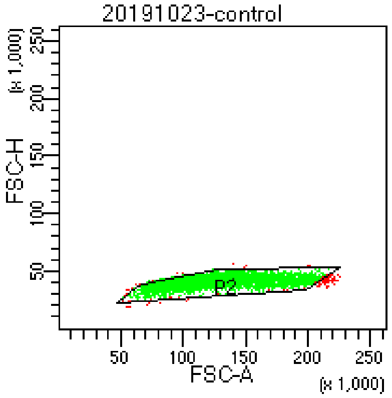

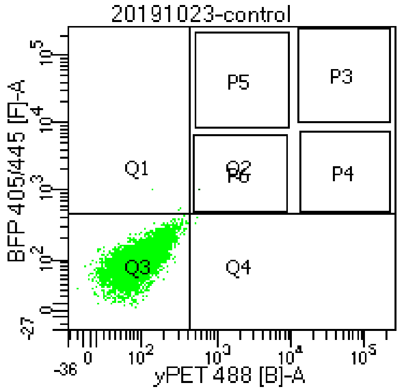

Tube: control

Population	#Events	%Parent	%Total
All Events	16,056	####	100.0
P1	10,321	64.3	64.3
P2	10,167	98.5	63.3
Q1	3	0.0	0.0
Q2	1	0.0	0.0
Q3	10,160	99.9	63.3
Q4	3	0.0	0.0
P3	0	0.0	0.0
P4	0	0.0	0.0
P5	0	0.0	0.0
P6	1	0.0	0.0

BD FACSDiva 8.0.1

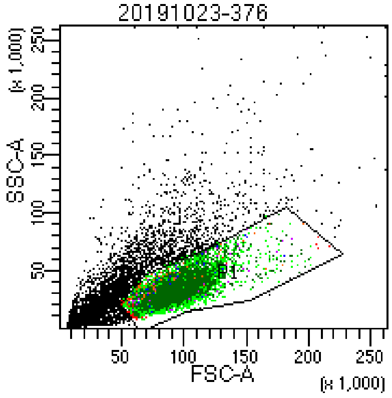

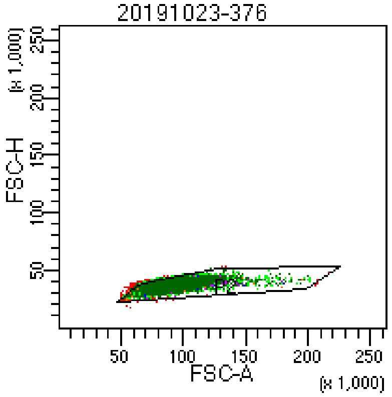

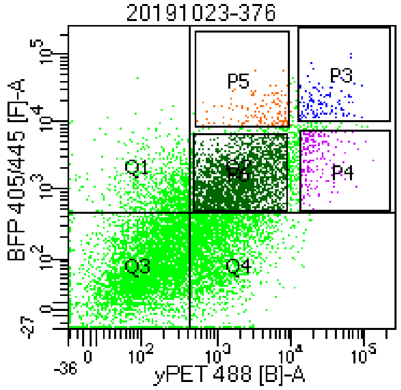

Tube: 376

Population	#Events	%Parent	%Total
All Events	15,517	####	100.0
P1	11,262	72.6	72.6
P2	11,040	98.0	71.1
Q1	802	7.3	5.2
Q2	3,098	28.1	20.0
Q3	4,839	43.8	31.2
Q4	2,301	20.8	14.8
P3	124	1.1	0.8
P4	193	1.7	1.2
P5	139	1.3	0.9
P6	2,193	19.9	14.1

Printed on: Wed Oct 23, 2019 04:32:39 CEST

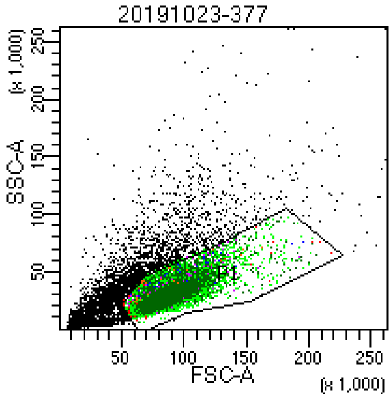

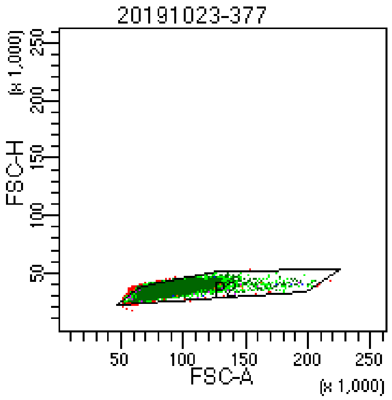

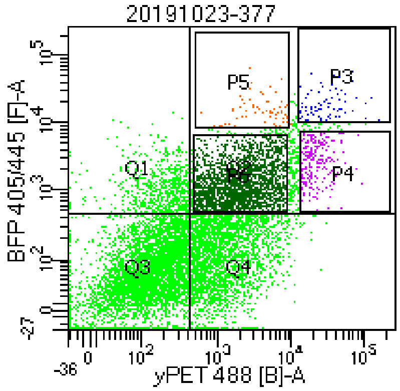

Tube: 377

Population	#Events	%Parent	%Total
All Events	15,022	####	100.0
P1	11,017	73.3	73.3
P2	10,828	98.3	72.1
Q1	697	6.4	4.6
Q2	2,789	25.8	18.6
Q3	4,792	44.3	31.9
Q4	2,550	23.6	17.0
P3	81	0.7	0.5
P4	242	2.2	1.6
P5	70	0.6	0.5
P6	1,997	18.4	13.3

BD FACSDiva 8.0.1

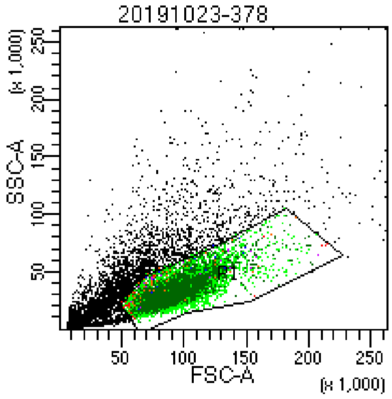

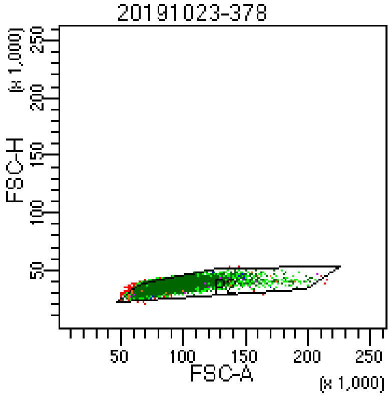

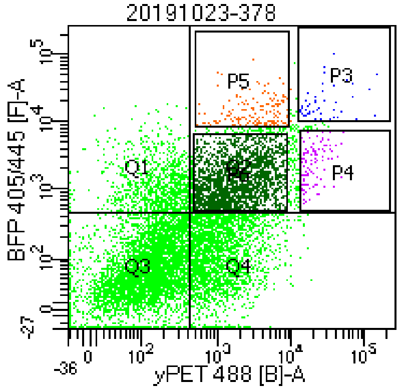

Tube: 378

Population	#Events	%Parent	%Total
All Events	14,541	####	100.0
P1	10,809	74.3	74.3
P2	10,661	98.6	73.3
Q1	983	9.2	6.8
Q2	2,673	25.1	18.4
Q3	4,663	43.7	32.1
Q4	2,342	22.0	16.1
P3	50	0.5	0.3
P4	115	1.1	0.8
P5	161	1.5	1.1
P6	2,000	18.8	13.8
