# Supplementary figures and images for: Smart-watch-programmed green-light-operated percutaneous control of therapeutic transgenes
Source: Nat Commun. 2021 Jun 7;12:3388. doi: 10.1038/s41467-021-23572-4 (PMC8184832; doi:10.1038/s41467-021-23572-4)

Original Western blots referring to Supplementary Figure 6c

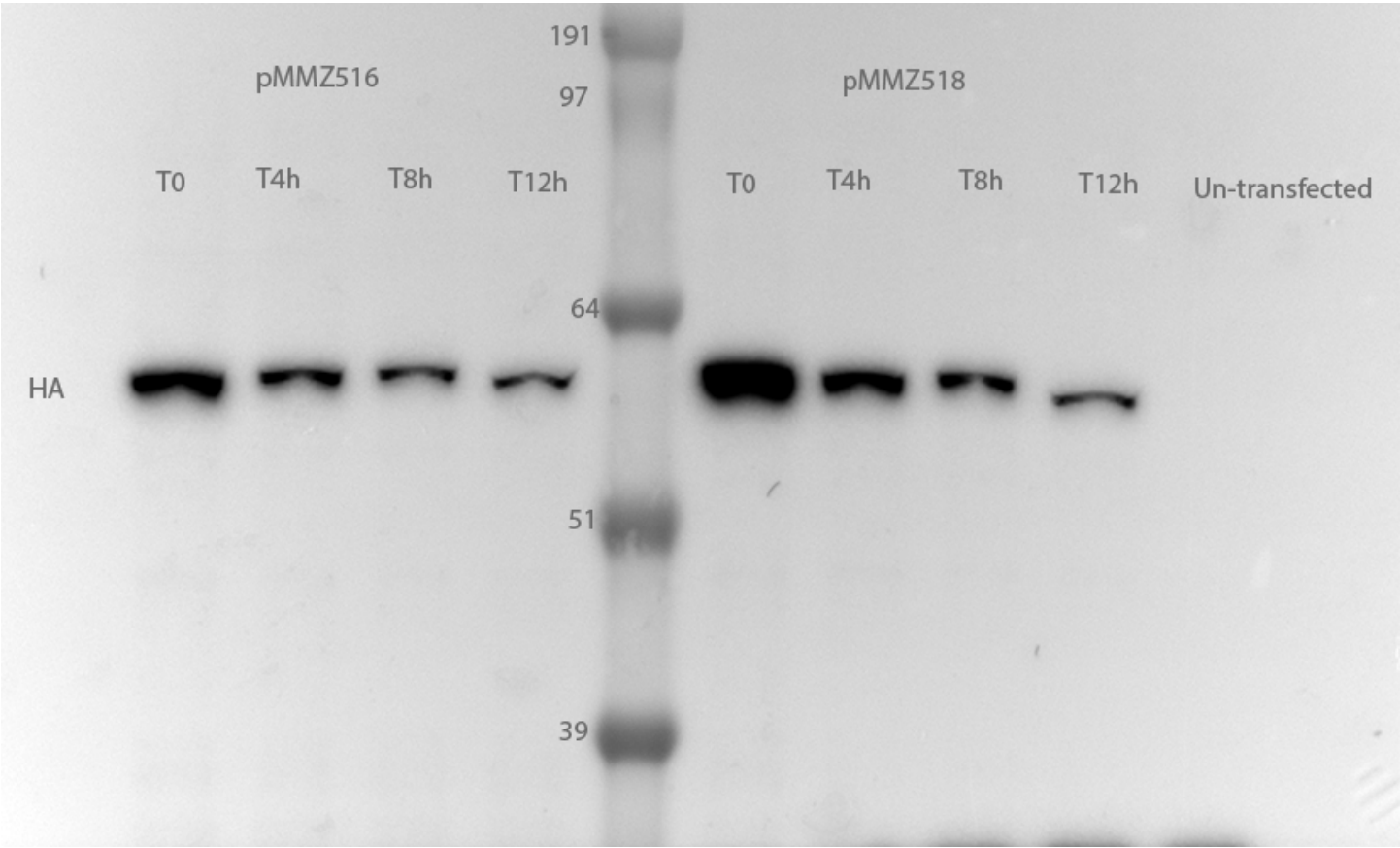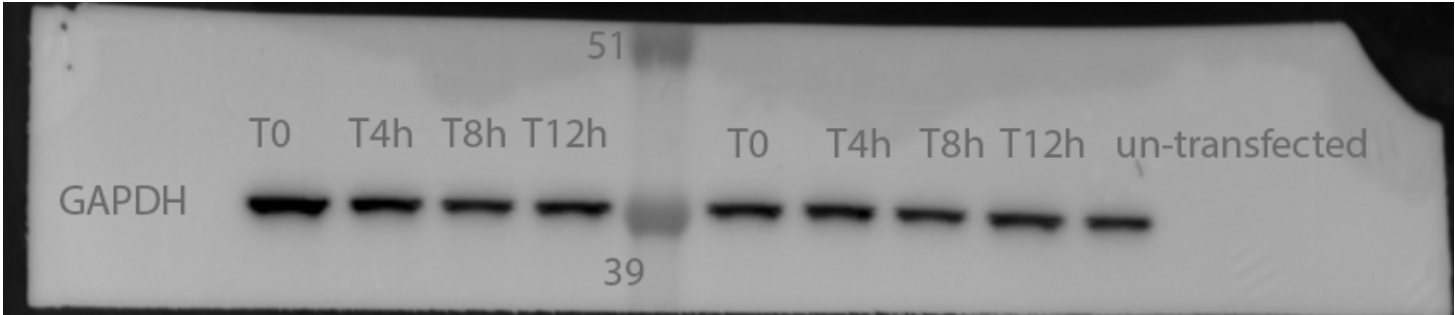

Supplement: Supplementary file 4 — Source Data [file 41467_2021_23572_MOESM4_ESM.zip › Data Source/Fig_SI_6c.pdf]
